# Impact of blood collection and processing on peripheral blood gene expression profiling in type 1 diabetes

**DOI:** 10.1186/s12864-017-3949-2

**Published:** 2017-08-18

**Authors:** Linda Yip, Rebecca Fuhlbrigge, Mark A. Atkinson, C. Garrison Fathman

**Affiliations:** 1 Department of Medicine, Division of Immunology and Rheumatology, Stanford University, Stanford, CA 94305 USA; 2 Department of Pathology, Immunology and Laboratory Medicine, University of Florida, Gainesville, FL 32610 USA

**Keywords:** Tempus, PAXgene, Peripheral blood, Microarray, Gene expression, Type 1 diabetes, NanoString, RNA isolation

## Abstract

**Background:**

The natural history of type 1 diabetes (T1D) is challenging to investigate, especially as pre-diabetic individuals are difficult to identify. Numerous T1D consortia have been established to collect whole blood for gene expression analysis from individuals with or at risk to develop T1D. However, with no universally accepted protocol for their collection, differences in sample processing may lead to variances in the results. Here, we examined whether the choice of blood collection tube and RNA extraction kit leads to differences in the expression of genes that are changed during the progression of T1D, and if these differences could be minimized by measuring gene expression directly from the lysate of whole blood.

**Results:**

Microarray analysis showed that the expression of 901 genes is highly influenced by sample processing using the PAXgene versus the Tempus system. These included a significant number of lymphocyte-specific genes and genes whose expression has been reported to differ in the peripheral blood of at-risk and T1D patients compared to controls. We showed that artificial changes in gene expression occur when control and T1D samples were processed differently. The sample processing-dependent differences in gene expression were largely due to loss of transcripts during the RNA extraction step using the PAXgene system. The majority of differences were not observed when gene expression was measured in whole blood lysates prepared from blood collected in PAXgene and Tempus tubes.

**Conclusion:**

We showed that the gene expression profile of samples processed using the Tempus system is more accurate than that of samples processed using the PAXgene system. Variation in sample processing can result in misleading changes in gene expression. However, these differences can be minimized by measuring gene expression directly in whole blood lysates.

**Electronic supplementary material:**

The online version of this article (doi:10.1186/s12864-017-3949-2) contains supplementary material, which is available to authorized users.

## Background

Type 1 Diabetes (T1D) is characterized by the gradual autoimmune destruction of insulin-producing pancreatic beta cells. It is the most common autoimmune disease in children, with approximately 1 in 500 affected in the USA [1]. However, the disorder’s pathogenesis and natural history remains unclear due, in part, to the difficulty in identifying and obtaining samples from at-risk individuals prior to the onset of hyperglycemia, and the lack of validated biomarkers of the disease. To fill the void and meet this need, various multicenter T1D consortia have been established to screen and follow susceptible individuals and recently diagnosed subjects, and to collect biological samples from such persons (e.g., peripheral blood cells (PBC)). For example, the NIH TrialNet Pathway to Prevention study, NIH Environmental Determinants of Diabetes in the Young study (TEDDY), Diabetes Autoimmune Study in the Young (DAISY), and Type 1 Diabetes Prediction and Prevention Project (DIPP) have collectively enrolled more than 20,000 individuals with an increased risk of developing T1D. The samples provided by these repositories represent a valuable resource, allowing researchers to control, at a geographically diverse level, for ethnic or environmental factors that might confound samples collected at a single center.

Whole blood gene expression analysis may be used to identify biomarkers of disease risk and progression and to potentially help characterize the pathogenesis of T1D [2–4]. Developing diagnostic or prognostic tools based on peripheral blood gene expression is particularly appealing since blood is easily obtained and requires minimal processing. PBC RNA is considered stable for extended periods of time when stored in either PAXgene or Tempus RNA tubes [5]. These tubes contain reagents that stabilize RNA and prevent induction or degradation of transcripts that normally would occur within minutes of blood collection in conventional tubes [5–7]. PAXgene tubes (used by TEDDY, DAISY, and DIPP) contain tetradecyl trimethyl-ammonium oxalate and tartaric, while Tempus tubes (used by TrialNet) contain detergent and guanidine. Both solutions immediately lyse cells, inactivate RNases, and precipitate RNA [2, 4, 8–10]. RNA can then be extracted using kits that are optimized for each collection tube [11]. Previous studies have shown that PBC gene expression can be influenced by the choice of collection tube and/or the RNA extraction system used [12–16]. However, it is unclear which system most accurately reflects the true gene expression profile and if sample-processing-related differences might mask the true gene expression profile or result in artifactual changes in gene expression. It is also unclear if sample-processing dependent differences in gene expression could be minimized by omitting the RNA extraction step. This is possible by measuring gene expression directly from whole blood lysate using NanoString arrays. We believe these represent important issues for any gene expression profiling study utilizing both Tempus and PAXgene-processed samples. Here, we examined the impact of sample collection and processing on PBC gene expression analysis in T1D.

## Methods

**Fig. 1:**
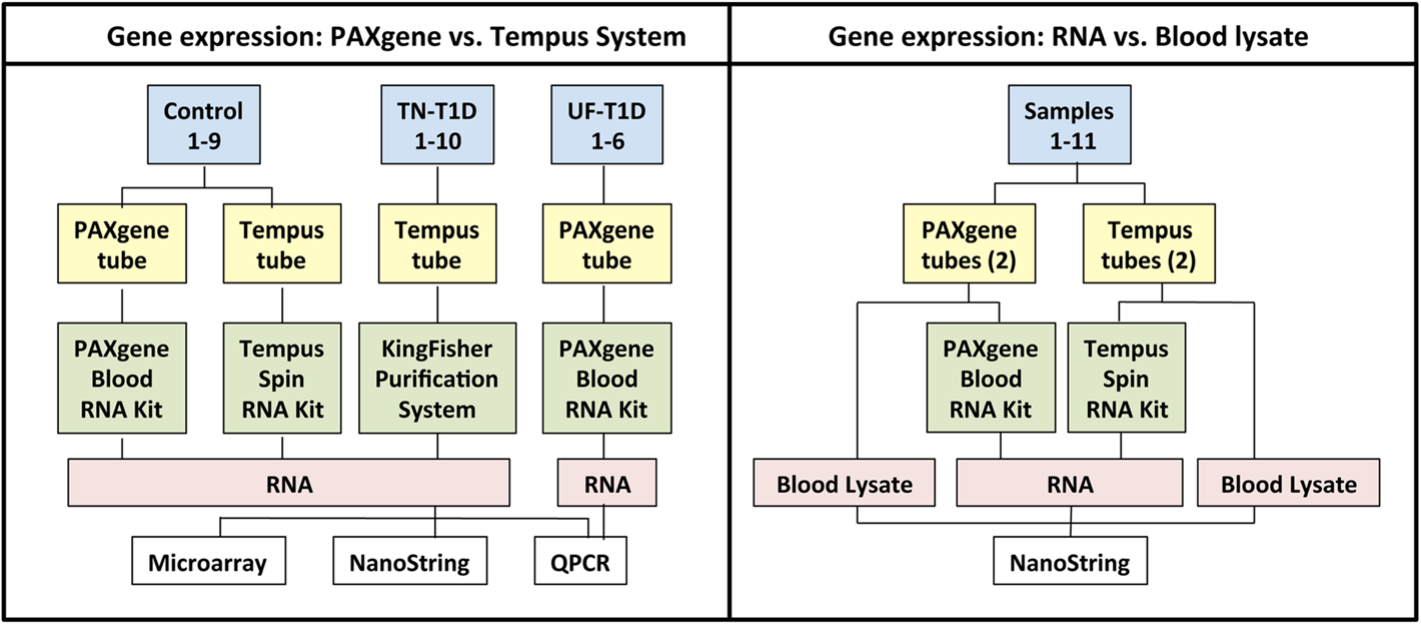
Study design. *Left-hand panel:* To identify genes that are differentially expressed in whole blood samples collected and processed using the PAXgene versus Tempus systems, matching samples of whole blood were collected from 9 healthy individuals (Control 1–9). Samples were processed using either the PAXgene Blood RNA Kit or the Tempus Spin RNA Kit. Gene expression was analyzed by microarray analysis and compared. To examine if differences in sample processing can result in artificial changes in gene expression between healthy and diseased individuals, we compared gene expression in samples from T1D subjects that were obtained from TrialNet (TN-T1D) and the University of Florida (UF-T1D) to that of healthy subjects (Control 1–9). TrialNet samples were collected in Tempus tubes and RNA was isolated using the automated KingFisher Purification system. University of Florida samples were collected in PAXgene tubes and processed using the PAXgene blood RNA kit. *Right-hand panel*: To examine if sample processing-dependent differences in gene expression are due to degradation of transcripts in the collection tube or loss of transcripts during the RNA extraction procedure, we compared gene expression in matching samples of isolated RNA versus whole blood lysate prepared directly from the collection tubes. For this study, four whole blood samples were collected from 11 healthy individuals (Samples 1–11), and blood lysate and total RNA were prepared from 1 PAXgene tube and 1 Tempus tube each

To compare gene expression in whole blood samples processed using the PAXgene vs. Tempus systems and in isolated RNA vs. whole blood lysates, blood was collected from healthy non-T1D related individuals at Stanford University. Samples from established T1D patients were collected by TrialNet or the University of Florida (Table 1 and Fig. 1). Approval was obtained from the Stanford University Institutional Review Board (IRB) or the University of Florida IRB. For TrialNet samples (Study TN08), IRB approval was obtained at the institution where samples were collected. All participants provided written informed consent.

**Table 1:** Sample information

ID	Age	Sex	RNA Yield (ng/ml)		PAXgene yield (% Tempus)	RIN	
			PAXgene	Tempus		PAXgene	Tempus
Normal controls in PAXgene and Tempus tubes for RNA extraction							
Control-1	73	M	5510	6572	84	7.0	7.5
Control-2	33	M	1082	1339	81	8.0	7.9
Control-3	37	M	3262	3598	91	7.4	7.7
Control-4	51	M	3716	2279	163	7.1	9.1
Control-5	26	M	3826	3324	115	7.8	8.8
Control-6	63	M	1071	2636	41	7.7	7.6
Control-7	32	M	1007	1858	54	7.8	9.5
Control-8	30	M	3676	3930	94	7.0	8.5
Control-9	40	F	4669	5772	81	7.7	7.5
TrialNet type 1 diabetes patient samples in Tempus tubes							
ID	Age	Sex	Duration (y)		Serum Auto-antibodies^a^		RIN
TN-T1D1	23	M	2.25		GAD65, ICA512, mIAA, ICA		7.4
TN-T1D2	23	F	2.25		GAD65, ICA512, mIAA		8.4
TN-T1D3	26	F	2.28		GAD65, mIAA		7.8
TN-T1D4	13	F	2.26		ICA512, mIAA, ICA		8.5
TN-T1D5	40	M	2.22		GAD65, ICA512, mIAA		8.6
TN-T1D6	18	F	2.26		GAD65, mIAA		8.2
TN-T1D7	18	M	2.24		GAD65, ICA512, mIAA		7.6
TN-T1D8	14	F	2.24		GAD65, mIAA		7.6
TN-T1D9	16	F	2.19		ICA512, mIAA, ICA		7.3
TN-T1D10	16	M	2.28		GAD65, ICA512, mIAA		8.3
University of Florida type 1 diabetes patient samples in PAXgene tubes							
ID	Age	Sex	Duration (y)		Serum Auto-antibodies^a^		RIN
UF-T1D1	17	F	2.67		GADA, IA-2		9.7
UF-T1D2	16	M	2.42		Not detected		7.1
UF-T1D3	16	M	2.00		GADA, IA-2, ZnT8		9.1
UF-T1D4	19	F	2.33		GADA		7.9
UF-T1D5	17	F	2.25		GADA, IA-2, ZnT8		8.8
UF-T1D6	27	M	1.92		GADA, IA-2, ZnT8		8.0
Normal controls for RNA extraction and lysate preparation							
ID	Age	Sex	RNA Yield (ng/ml)		PAXgene yield (% Tempus)	RIN	
			PAXgene	Tempus		PAXgene	Tempus
Sample 1	19	M	1508	4690	32	6.3	9.0
Sample 2	19	M	947	3088	31	8.4	8.8
Sample 3	20	F	1441	4897	29	8.6	8.0
Sample 4	19	F	2982	3383	88	8.7	8.6
Sample 5	19	M	2604	3249	80	9.2	8.6
Sample 6	24	M	2016	905	223	8.2	8.7
Sample 7	20	F	2112	2942	72	9.2	7.0
Sample 8	24	F	3218	3769	85	8.5	8.2
Sample 9	19	M	1013	1916	53	8.2	8.7
Sample 10	29	F	3411	4031	85	8.2	8.7
Sample 11	26	M	5129	3923	131	7.5	9.0

^a^ *TN-T1D samples were screened for the following autoantibodies: Glutamic acid decarboxylase 65 (GAD65), Islet cell autoantigen 512 (ICA512, also referred to as IA-2), micro Insulin autoantibodies (mIAA), and islet cell cytoplasmic autoantibodies (ICA)*. *UF-T1D samples were screened for autoantibodies against glutamic acid decarboxylase (GADA), IA-2, and zinc transporter 8 (ZNT8)*

### Sample collection and RNA extraction

To compare gene expression in PAXgene vs. Tempus-processed samples, whole blood of 9 healthy individuals was collected into both PAXgene (Preanalytix) and Tempus (Applied Biosystems) tubes (Table 1 and Fig. 1). Total RNA was extracted using the PAXgene Blood RNA Kit (Qiagen) or the Tempus Spin RNA Isolation Kit (Applied Biosystems), as appropriate, according to manufacturer’s instructions. TrialNet T1D (TN-T1D; Table 1) samples were collected in Tempus tubes and RNA was extracted by TrialNet using the KingFisher Purification system (Thermo Scientific). University of Florida T1D samples (UF-T1D; Table 1 and Fig. 1) were collected in PAXgene tubes and isolated using the PAXgene Blood RNA Kit (Qiagen). Total RNA concentrations were determined using the Nanodrop (Thermo Fisher) and RNA quality was assessed using the 2100 Bioanalyzer and the RNA 6000 Nano Reagent Kit (Agilent).

### Whole blood lysate preparation

To compare gene expression in matching samples of total RNA and whole blood lysate, 4 blood samples were collected from each of 11 control subjects, 2 into PAXgene and 2 into Tempus tubes (Table 1 and Fig. 1). RNA was extracted from a single PAXgene and Tempus tube as described above. Whole blood lysates were prepared from the remaining 2 tubes as follows: PAXgene samples were pelleted, washed and dried according to manufacturer’s instructions, and resuspended in 150 μl Buffer RLT (Qiagen) containing β-mercaptoethanol. Tempus samples were diluted in PBS and pelleted according to manufacturer’s instructions. RNA pellets were dried and resuspended in 50 μl of the same buffer.

### Microarray analysis

One color microarray analysis was performed at the Stanford Human Immune Monitoring Center using the SurePrint G3 Human Gene expression 8×60K one-color microarray kit and the Low Input QuickAmp Labeling Kit (Agilent). This system utilizes OligodT-promoter primer and T7 RNA polymerase to generate cRNA, and produces reliable and consistent gene expression results without globin removal, a procedure that substantially reduces the quantity and quality of extracted RNA [13, 14]. Total RNA (300 ng) containing 2 μl of spike-in control was amplified, fluorescently labeled, and hybridized to the microarray chips according to manufacturer’s instructions. Data were processed with Feature Extraction Software (version 12.0, Agilent), quantile-normalized and analyzed using GeneSpring GX 12.6 (Agilent). To compare gene expression between PAXgene and Tempus RNA samples, samples were filtered for detected entities (detected in ≥5 of 9 samples, Table 1, Control 1–9). Entities representing 17,843 unique genes that were detected using both collection tubes were further analyzed for differential gene expression by performing a moderated T-test with Benjamini-Hochberg multiple testing correction (*P* < 0.05) with a fold-change cut-off of 2. Hierarchical clustering (Euclidean Distance Metric, Ward’s Linkage Rule) was performed using GeneSpring GX 12.6.

To compare TN-T1D samples against Tempus controls (Control 1–9), gender-specific genes (see Additional file 1) were first omitted due to the imbalance of male to female subjects in the two groups. Gender-specific genes were identified by comparing male to female samples in the Tempus control and TN-T1D groups (Moderated T-test, Benjamini-Hochberg multiple testing correction, *P* < 0.05). Differentially expressed genes in TN-T1D vs. healthy Tempus controls were identified, and statistical analysis and hierarchical clustering were performed as described above. Enrichment of differentially expressed genes for B-cell, T-cell, granulocyte, and lymphocyte signature genes was examined using previously published lists of cell-type specific genes [17]. Significant enrichment was determined using the Chi-squared test with Yates’ correction (two-tailed, *P* < 0.05). All microarray data are available at NCBI Gene Expression Omnibus (GEO) Database (GSE89021 and GSE89022).

### QPCR

First strand cDNA was generated using Superscript III Supermix, containing a mixture of random hexamers and Oligo(dT)_20_ (Invitrogen). QPCR was performed using the 7900HT Fast Real Time PCR System, Taqman Gene Expression Mastermix and Taqman gene expression assays (all Applied Biosystems) as previously described [18]. Genes that were changed by at least 2-fold in PAXgene vs. Tempus RNA samples (*COX6C, COX7B, COMMD6, LSM3, RPS24, UQCRB and RPL31)*, and in TN-T1D vs. Tempus controls (*FASLG, FCRL6, GZMB,* and *KLRD1*) were measured, along with *GUSB,* a stably expressed housekeeping gene [19]. QPCR data were normalized using the housekeeping gene *ACTB.* The comparative Ct method (ΔΔCt) was used for relative quantification, and statistical analysis was performed using the Wilcoxon-matched pairs test or the Mann-Whitney test, where appropriate (*P* < 0.05).

### NanoString arrays using total RNA and blood lysate

NanoString arrays were used to assess the expression of genes in total RNA and whole blood lysate samples (Table 1). Custom probes were designed by NanoString based on the microarray probes for *COMMD6, COX6C, COX7B, FASLG, FCRL6, GZMB, IGFBP3, KIAA1841, KIF20B, KLRD1, LSM3, RPS24, SUB1, ZNF680, GUSB* (Table 2). Gene expression was measured using 200 ng total RNA or 1.5 μl blood lysate with the nCounter Master Kit, nCounter Prep Station (GEN1) and Digital analyzer (NanoString Techonologies), as described by the manufacturer. Data were analyzed with nSolver Analysis Software (version 2.6, NanoString Technologies). Raw counts were obtained and background subtraction was performed using the geometric mean of the negative controls. Data was normalized using the geometric mean of the positive control samples and *GUSB* housekeeping gene expression*.* Statistics were performed using the Wilcoxon-matched pairs test or Mann-Whitney test, where appropriate (*P* < 0.05).

**Table 2:** NanoString Probe Sequences

Gene	Sequence
*COMMD6*	CTTACGTTGCAGTGATGCTAAAAGTGGCAGATCATTCAGGCCAAGTAAAGACCAAGTGCTTTGAAATGACGATTCCACAGTTTCAGAATTTCTACAGACA
*COX6C*	GCTTTGTATAAGTTTCGTGTGGCTGATCAAAGAAAGAAGGCATACGCAGATTTCTACAGAAACTACGATGTCATGAAAGATTTTGAGGAGATGAGGAAGG
*COX7B*	CAGAGCCACCAGAAACGTACACCTGATTTTCATGACAAATACGGTAATGCTGTATTAGCTAGTGGAGCCACTTTCTGTATTGTTACATGGACATATGTAG
*FASLG*	GCATATCCTGAGCCATCGGTGAAACTAACAGATAAGCAAGAGAGATGTTTTGGGGACTCATTTCATTCCTAACACAGCATGTGTATTTCCAGTGCAATTG
*FCRL6*	TGGAGAGCAGTGCCCACTATATGCCAACGTGCATCACCAGAAAGGGAAAGATGAAGGTGTTGTCTACTCTGTGGTGCATAGAACCTCAAAGAGGAGTGAA
*GZMB*	ATGGCATGCCTCCACGAGCCTGCACCAAAGTCTCAAGCTTTGTACACTGGATAAAGAAAACCATGAAACGCTACTAACTACAGGAAGCAAACTAAGCCCC
*IGFBP3*	GTCAGCCTCCACATTCAGAGGCATCACAAGTAATGGCACAATTCTTCGGATGACTGCAGAAAATAGTGTTTTGTAGTTCAACAACTCAAGACGAAGCTTA
*KIAA1841*	AAAGAGAAGACGATCAACGGCGAATGACTGAAATTACAGGGCACCTAATAAAAATGAGATTGGGGGATCTGGACCGAGTCAAGTCAAAGGAAGCAAAAGA
*KIF20B*	AATGGCAGTGAAACACCCTGGTTGTACCACACCAGTGACAGTTAAGATTCCCAAGGCTCGGAAGAGGAAGAGTAATGAAATGGAGGAGGACTTGGTGAAA
*KLRD1*	GAAAAATTCTTTTACTAAACTGAGTATTGAGCCAGCATTTACTCCAGGACCCAACATAGAACTCCAGAAAGACTCTGACTGCTGTTCTTGCCAAGAAAAA
*LSM3*	TTGAAACATGGCGGACGACGTAGACCAGCAACAAACTACCAACACTGTAGAGGAGCCCCTGGATCTTATCAGGCTCAGCCTAGATGAGCGAATTTATGTG
*RPS24*	TTGGTGGTGGCAAGACAACTGGCTTTGGCATGATTTATGATTCCCTGGATTATGCAAAGAAAAATGAACCCAAACATAGACTTGCAAGACATGGCCTGTA
*SUB1*	GGCAAAGTGCTAATTGATATTAGAGAATATTGGATGGATCCTGAAGGTGAAATGAAACCAGGAAGAAAAGGTATTTCTTTAAATCCAGAACAATGGAGCC
*ZNF680*	GGCCTCCCTAAAGATGTGGGATTACAGAAATAATCCACTTTGCCAGGCTACTATGTAGCCCTTCATGTAGAACTGTGCTAGTCATAATGAATCATAACAC
*GUSB*	GACTGTTCACGGCAGACCAGAACGTTTCTGGCCTGGGTTTTGTGGTCATCTATTCTAGCAGGGAACACTAAAGGTGGAAATAAAAGATTTTCTATTATGG

## Results

### Gene expression in samples processed using the PAXgene versus tempus system

**Fig. 2:**
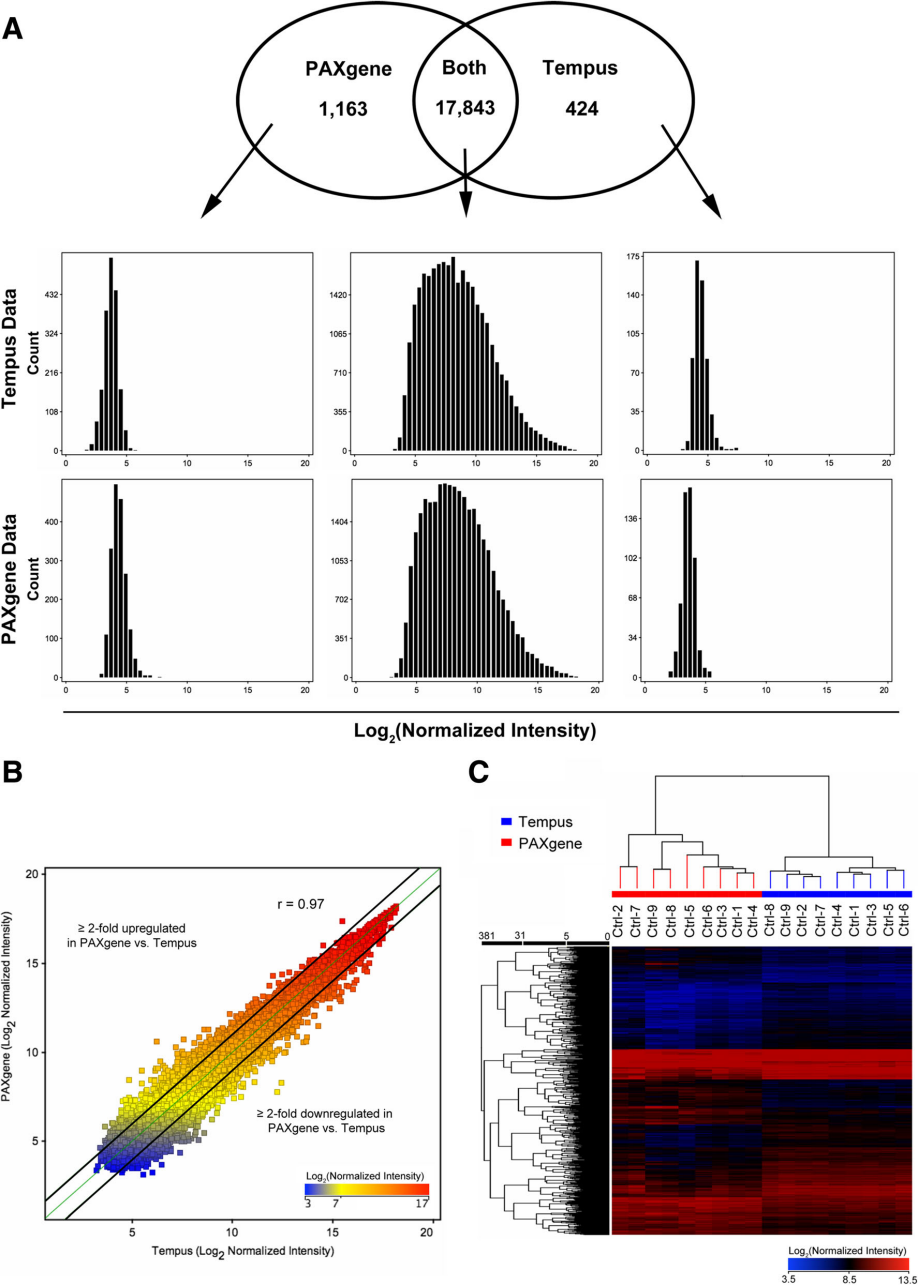
Gene expression in blood samples collected and processed using the PAXgene versus Tempus system. **a** Venn-diagram showing the number of genes that are expressed in the majority of samples (≥5 of 9 samples) collected and processed using either the PAXgene or Tempus system. Histograms show the Log_2_(normalized intensity) of genes in each group. Genes detected using both systems (*middle panels*) contain the most abundantly expressed genes, while genes that are detected using one system but not the other (*left* and *right panels*) are among the 10% of genes with the lowest expression levels. **b** Scatter plot showing significant correlation between the expression of genes measured in PAXgene-processed and Tempus-processed samples. Dots outside the black lines represent the 5% of genes (901 unique genes) that are upregulated (1.5%; 287 genes) or downregulated (3.5%; 614 genes) by ≥2-fold in samples processed using the PAXgene vs. Tempus system (*P* < 0.05; Moderated T-test with Benjamini-Hochberg correction). **c** Heatmap showing the hierarchical clustering of samples based on genes that are differentially expressed by at least 2-fold

**Fig. 3:**
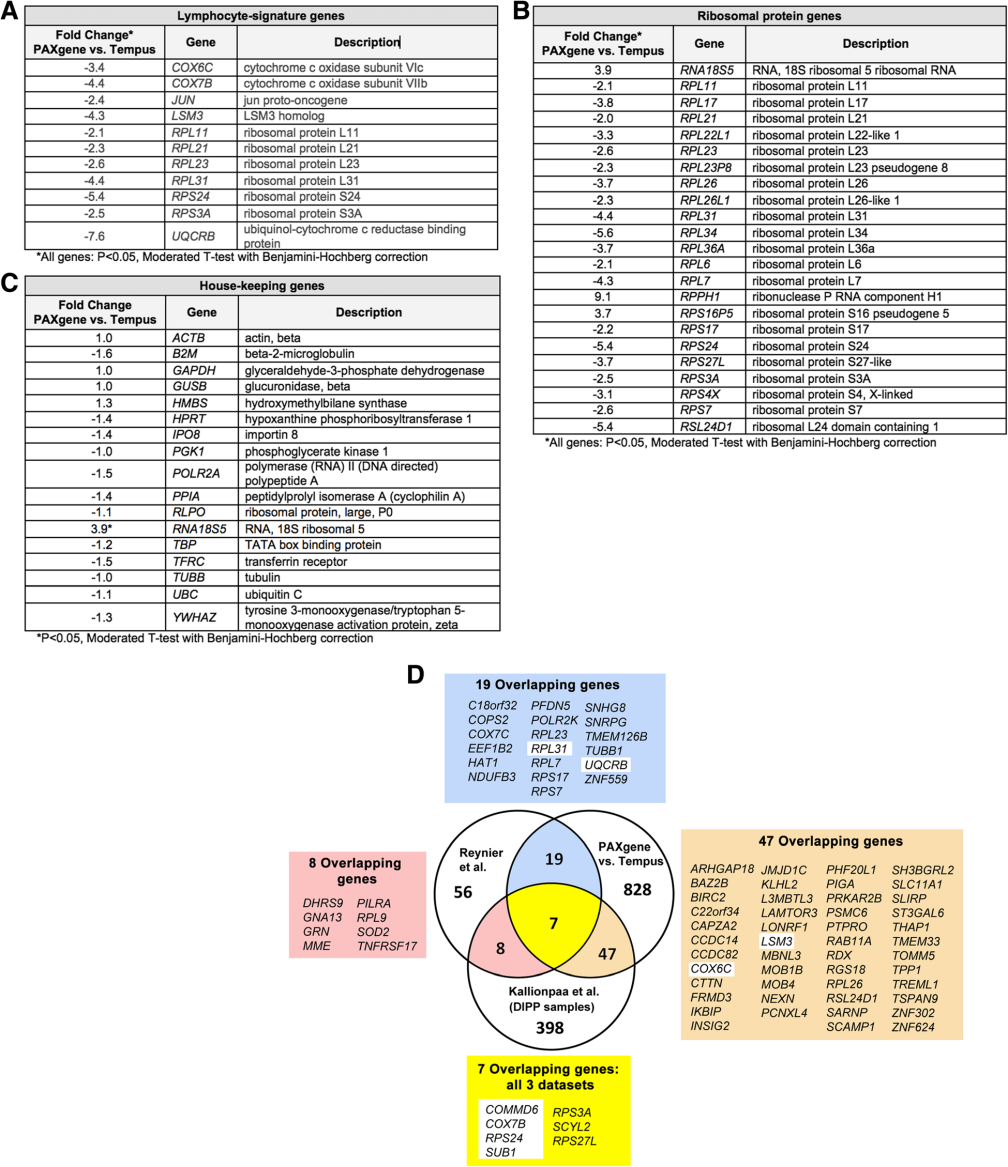
Sample processing-dependent changes in PBC gene expression. Sample processing-dependent genes were highly enriched for lymphocyte-signature genes (**a**), ribosomal protein genes (**b**), and genes that have previously been reported to change in the peripheral blood of at-risk or T1D patients (**d**, [3, 4]). The majority of housekeeping genes were not affected by sample processing (**c**). The Venn-diagram (**d**) shows that ~29% (26 out of 90 on the microarray) of genes shown by Reynier et al. to be differentially expressed in at-risk AA+ first-degree relatives of T1D patients and T1D patients at clinical onset, compared to healthy controls and 12% (54 out of 460 on the microarray) of genes found by Kallionpaa et al. that are changed in the whole blood of T1D progressors compared to healthy controls overlap with the 901 sample-processing dependent genes in our study [3, 4]. Genes highlighted in white in panel **d** were further validated by QPCR and/or NanoString arrays (see Fig. 3)

The amounts of RNA recovered from whole blood samples collected in PAXgene and Tempus tubes were similar and within previously reported ranges [13–15, 20]. RNA quality was also comparable (Table 1). All samples had a RNA integrity number (RIN) of ≥7.0 and an A260/A280 ratio between 2.00–2.15. Microarray analysis showed that 17,843 unique genes, including the most abundantly expressed genes, were detected in the majority (≥5 of 9) of samples processed using both the PAXgene and Tempus systems (Fig. 2a). An additional 1163 and 424 genes that were expressed in low abundance were detected in most samples using either the PAXgene or Tempus system, respectively. These genes were among the 10% of genes with the lowest normalized intensity values on the microarray. Only 17 genes were detected in the majority of samples using one system, but not in any sample using the other (see Additional file 2). A Pearson correlation coefficient of 0.97 was observed between gene expression levels measured in PAXgene vs. Tempus-processed samples. Approximately 5% of genes (901 unique genes) were significantly changed by ≥2-fold in samples processed using the PAXgene vs. Tempus system (*P* < 0.05, Moderated T-test, Benjamini-Hochberg correction; Fig. 2b and c, see Additional file 2). Similar to previous reports, the majority of these genes (614, or 68%) were reduced in samples processed using the PAXgene vs. Tempus system [13]. These differentially expressed genes were significantly enriched in lymphocyte-signature genes and ribosomal protein genes (*P* < 0.0001, two-tailed Chi-square with Yates’ correction, Fig. 3a and b), but not in B-cell, T-cell, CD8+ T-cell or granulocyte-signature genes [17]. Most housekeeping genes were not significantly influenced by the collection or RNA extraction system used, but the widely-used housekeeping gene *RNA18S5* (eukaryotic 18S ribosomal RNA) was ~4-fold higher in PAXgene-processed samples 3C).

The 901 genes affected by sample processing were also significantly enriched for genes that have previously been reported to be changed in the PBC of recent onset T1D patients and at-risk individuals: 26 genes (29%) overlapped with the 90 genes (present on our array) found by Reynier and colleagues [3] to be altered in the PBC of autoantibody-positive first-degree relatives of T1D patients and T1D patients at clinical onset compared to healthy controls (*P* < 0.0001, two-tailed Chi-square with Yates’ correction, Fig. 3d). 54 genes (12%) overlapped with the 460 genes (present on our array) found by Kallionpaa and colleagues [4] to be changed in the PBC of individuals who later progressed to T1D compared to controls (*P* < 0.0001, two-tailed Chi-square with Yates’ correction, Fig. 3d). These overlapping genes include lymphocyte-signature genes *COX6C, COX7B, LSM3, RPS24, RPL31*, *RPS3A* and *UQCRB (*Fig. 3a and d *)*. 7 genes overlapped in all 3 datasets (*COMMD6, COX7B, RPS24, SUB2, RPS3A, SCYL2* and *RPS27L*).

**Fig. 4:**
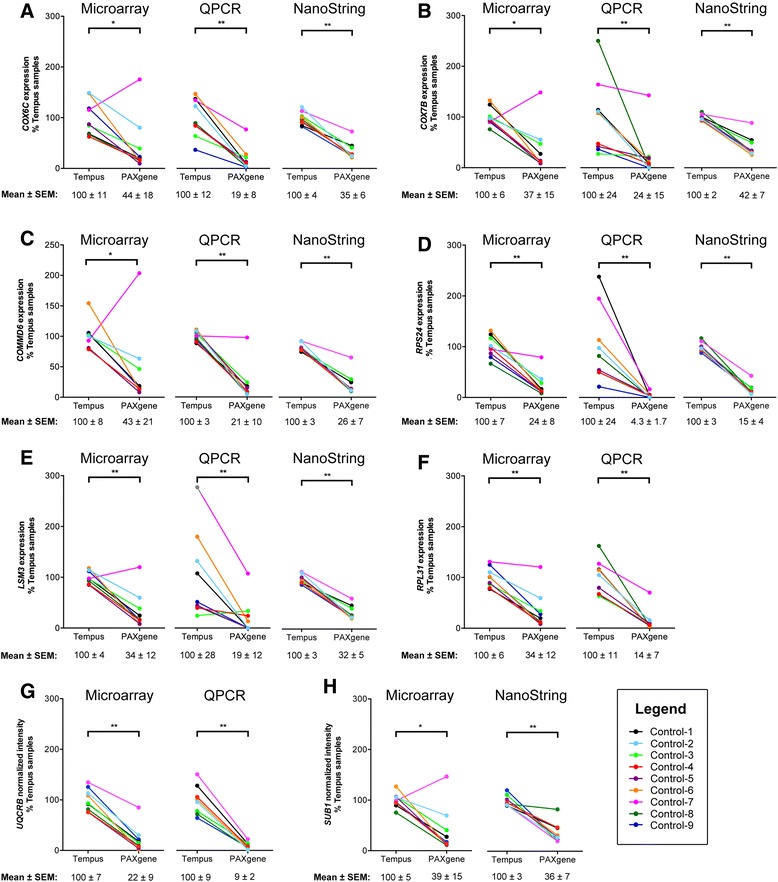
QPCR and NanoString arrays in matching PBC samples processed using the PAXgene and Tempus system. The expression of lymphocyte-signature genes (*COX6C, COX7B, LSM3, RPS24,* and *RPL31*), and genes previously shown to be changed in T1D or at-risk patients (*COX6C, COX7B, LSM3, RPS24, RPL31, COMMD6, SUB1,* and *UQCRB*; see Fig. 2) were measured by QPCR and/or NanoString arrays (**a**-**h**). Microarray, QPCR and/or NanoString arrays gave similar results, and NanoString array data showed the least amount of variation between samples. *P < 0.05; **P < 0.005 in PAXgene vs. Tempus-processed samples, two-tailed Wilcoxon matched-pairs signed rank test

We performed QPCR and/or NanoString arrays to validate the expression of *COX6C, COX7B, LSM3, RPS24, RPL31*, *UQCRB, COMMD6* and *SUB1* in healthy control samples processed using the PAXgene and Tempus systems (Fig. 4). Similar to microarray experiments, both QPCR and NanoString assays showed significantly reduced expression of all genes in RNA extracted using the PAXgene system. The NanoString arrays showed the least amount of variation between samples within a group, and consistently showed lower gene expression in all 9 PAXgene-RNA extracted samples compared to the matching Tempus-RNA extracted sample.

### Differential expression of sample-processing-dependent genes in the peripheral blood of established T1D patients

**Fig. 5:**
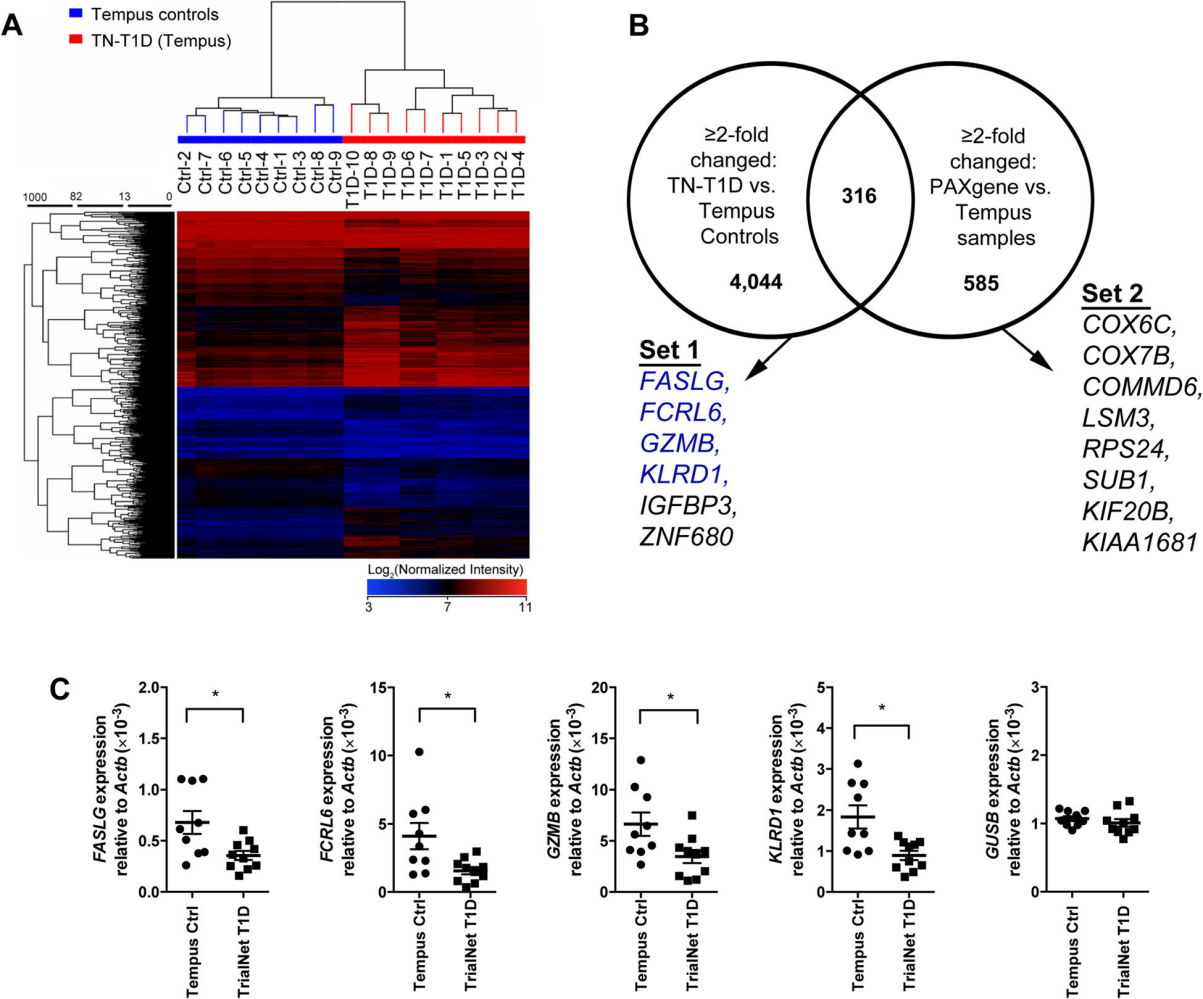
Differentially expressed genes in the peripheral blood of established T1D patients. **a** Heatmap showing the hierarchical clustering of samples based on genes that were found by microarray analysis to be differentially expressed by ≥2-fold in established TN-T1D patients compared to healthy controls (Moderated T-test, Benjamini-Hochberg correction, *P* < 0.05). Both TN-T1D and control samples were collected and processed in Tempus tubes. **b** Venn-diagram showing that 316 genes out of the 901 genes that are affected by sample processing are also differentially expressed in the peripheral blood of established T1D patients. The two sets of genes listed below were further examined by QPCR (in *blue*) and NanoString arrays (see Fig. 5). **c** QPCR data for select genes that are differentially expressed in TN-T1D patients compared to healthy controls (Tempus controls, *n* = 9; TN-T1D, *n* = 10; *P < 0.05, two-tailed unpaired Mann-Whitney test)

We performed microarray analysis on PBC RNA samples of 10 established T1D subjects obtained from TrialNet (TN-T1D, *n* = 10, disease duration ~2.3 years, collected in Tempus tubes and processed using the KingFisher purification system), and compared gene expression to the 9 control samples collected in Tempus tubes (see Table 1 and Fig. 1, Control 1–9). 20 gender-specific genes were identified and omitted from the analysis due to the imbalance of male to female subjects in the 2 groups (see Additional file 1). 4360 genes were found to be changed by ≥2-fold in TN-T1D samples compared to the Tempus controls. Expression of a significant number of genes (316 genes), overlapped with the 901 genes affected by sample processing (*P* < 0.0001, two-tailed Chi-square with Yates’ correction; Fig. 5a, b). We validated the expression of 4 genes (*FASLG, FCRL6, GZMB, and KLRD1)* by QPCR and showed reduced expression of all 4 genes in TN-T1D compared to Tempus controls (Fig. 5c). The housekeeping gene, *GUSB*, was not changed.

**Fig. 6:**
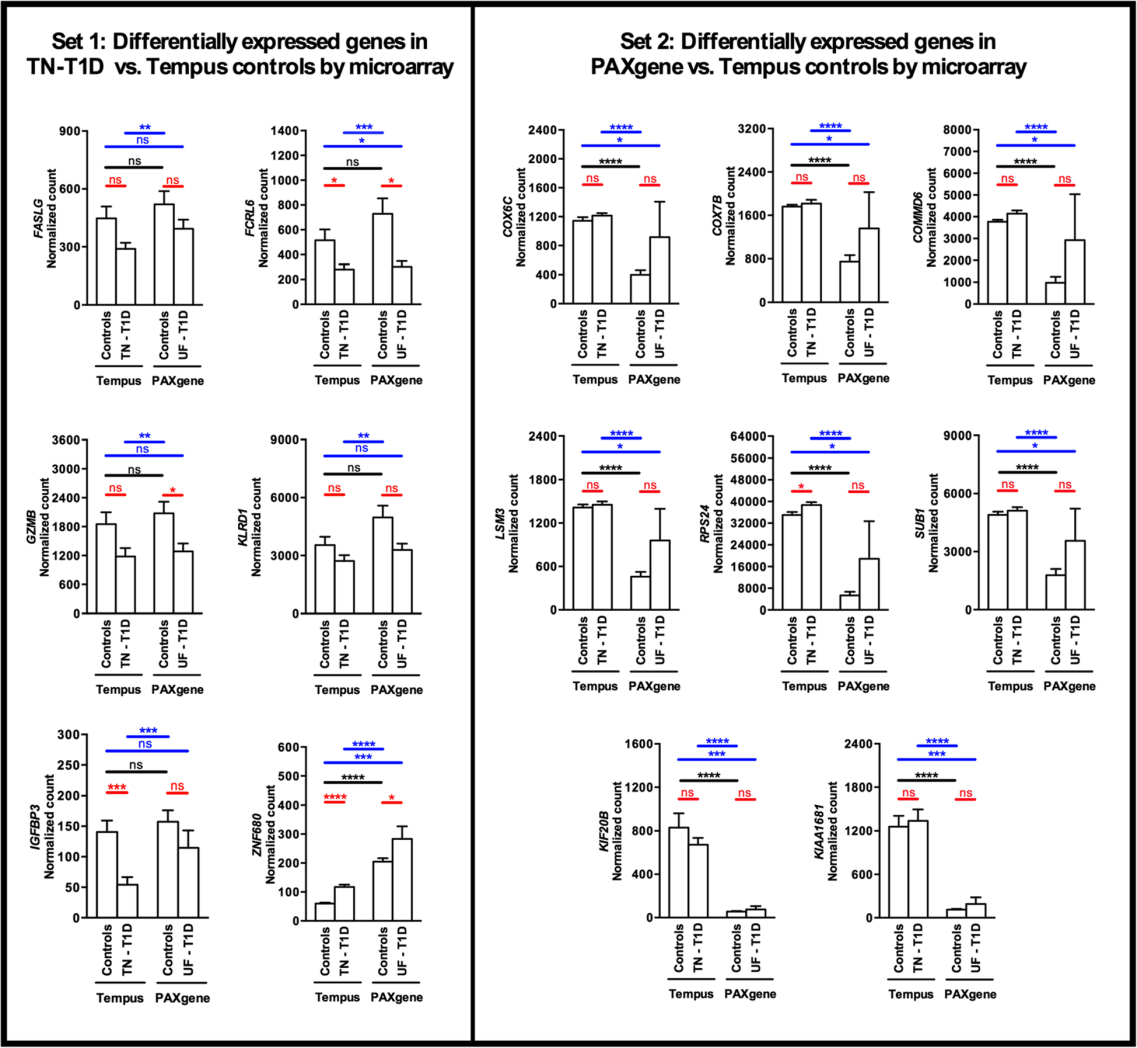
Gene expression in T1D and control samples processed using the Tempus and PAXgene systems. Two sets of genes were examined: genes found by microarray analysis to be differentially expressed in TN-T1D vs. Tempus controls (Set 1), and differentially expressed in control samples processed using the PAXgene vs. Tempus system (Set 2). NanoString arrays were performed to compare gene expression between TN-T1D (prepared in Tempus, *n* = 10), UF-T1D (prepared in PAXgene, *n* = 6), and healthy control samples (*n* = 9 prepared in Tempus; *n* = 9 prepared in Paxgene). *P < 0.05, **P < 0.01, ***P < 0.001, ****P < 0.0001, two-tailed unpaired Mann-Whitney test

NanoString assays were also performed to compare gene expression of T1D and control samples collected in Tempus and PAXgene tubes (Control 1–9 in Tempus and PAXgene tubes, TN-T1D 1–10 in Tempus tubes, UF-T1D 1–6 in PAXgene tubes, see Table 1). Two sets of genes were examined based on microarray data (Fig. 5b). The first consists of genes that were differentially expressed in TN-T1D compared to Tempus controls (Set 1: *FASLG, FCRL6, GZMB, KLRD1, IGFBP3* and *ZNF680*) and the second consists of genes that were differentially expressed in RNA samples extracted using the PAXgene vs. Tempus systems (Set 2: *COX6C, COX7B, COMMD6, LSM3, RPS24, SUB1, KIF20B,* and *KIAA1681*). NanoString results showed that all genes in the first set behaved as expected: expression of *FASLG, FCRL6, GZMB, KLRD1,* and *IGFBP3* were reduced while expression of *ZNF680* was increased in TN-T1D vs. Tempus controls and UF-T1D vs. PAXgene controls. The changes, however, were not all significant (Fig. 6; Set 1, comparisons in red). As expected, all genes in the second set were significantly reduced in control RNA samples processed using the PAXgene vs. Tempus system (Fig. 6; Set 2, comparisons in black) and were generally not significantly different between T1D samples and their respective controls (Fig. 6, Set 2, comparison in red). Significant changes in gene expression were observed if TN-T1D samples (collected in Tempus tubes) are compared to PAXgene controls and if UF-T1D samples (collected in PAXgene tubes) are compared to Tempus controls (Fig. 6, Set 2, comparisons in blue). These findings demonstrate that differences in blood collection and RNA extraction procedures between control and test subjects can result in artifactual changes in gene expression.

### Comparison of gene expression in blood lysate and extracted RNA

**Fig. 7:**
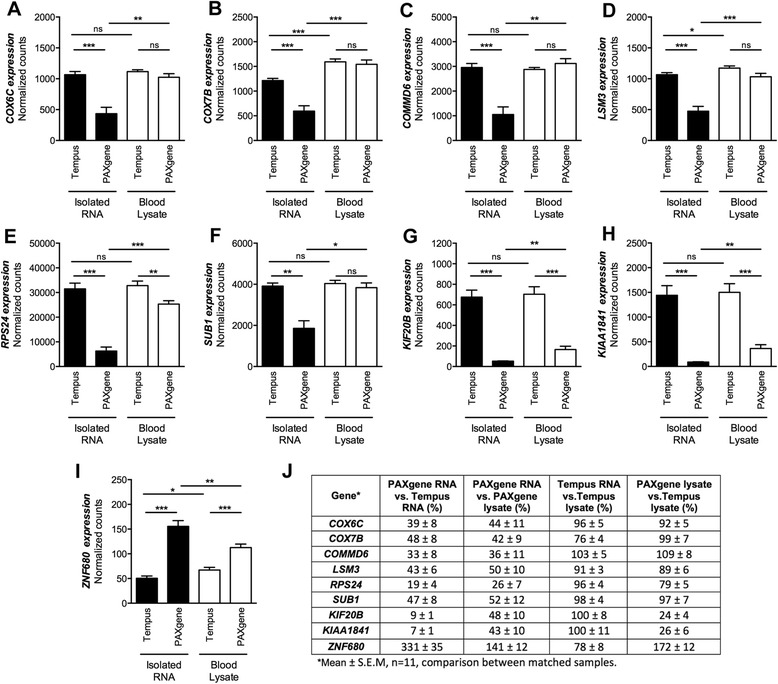
Comparison of gene expression in whole blood lysates and extracted RNA. **a-i** Genes that are affected by the collection tube or RNA extraction system (see Fig. 5) were measured by NanoString arrays in matching whole blood lysate and RNA samples prepared using the PAXgene or Tempus system (*n* = 11 per group). **j** Table showing relative levels of expression between groups. For all genes, expression was most similar between the Tempus whole blood lysate and Tempus RNA samples. The PAXgene lysate samples were also more similar to the Tempus lysate and Tempus RNA samples, than to the PAXgene RNA samples. *P < 0.05, **P < 0.01, ***P < 0.001, two-tailed Wilcoxon matched-pairs signed rank test

We next examined if the sample processing-dependent changes in gene expression we observed were due to degradation of certain gene transcripts while stored in the collection tube, or due to loss during the RNA extraction step. Matching blood samples were collected in PAXgene and Tempus tubes and gene expression was compared between minimally processed whole blood lysates vs. extracted total RNA in a separate group of 11 individuals (Table 1, Sample 1–11). We examined the expression of 9 genes that were found by NanoString arrays to be differentially expressed in the first cohort of 9 PAXgene vs. Tempus-processed RNA samples (Fig. 6). Similar to the first cohort, we showed that *COX6C, COX7B, COMMD6, LSM3, RPS24, SUB1, KIF20B,* and *KIAA1681* expression was significantly lower and *ZNF680* expression was significantly higher in RNA samples processed using the PAXgene system vs. the Tempus system (Fig. 7a-i, black bars). Similar fold-change differences in gene expression (PAXgene vs. Tempus) were observed for each of these genes in the first cohort (*n* = 9) and second cohort (*n* = 11) of individuals, suggesting that it may be possible to correct for sample-processing dependent differences in gene expression (see Additional file 3).

Comparison of the PAXgene and Tempus blood lysate samples showed that the expression of *COX6C, COX7B, COMMD6, LSM3,* and *SUB1* was not significantly different (Fig. 7a-d, f, *white* bars). This suggests that there is selective loss of these gene transcripts during the PAXgene RNA extraction procedure. *RPS24, KIF20B* and *KIAA1841* expression was still significantly different between PAXgene and Tempus whole blood lysates (Fig. 7e, g-h, *white* bars). However, the fold change differences were drastically lower. *KIF20B and KIAA1841* were approximately 11-fold and 14-fold lower in PAXgene vs. Tempus RNA samples, but only 4.1-fold and 3.8-fold lower in PAXgene vs. Tempus whole blood lysates, respectively (Fig. 7j). This suggests that a large amount of *KIF20B* and *KIAA1841* transcripts may have already degraded in the PAXgene collection tube prior to RNA extraction. However, some amount of *KIF20B* and *KIAA1841* was also lost during RNA extraction. Expression of *RPS24* was ~20% lower in PAXgene vs. Tempus lysate, and was 4-fold higher in PAXgene lysate vs. PAXgene RNA, suggesting that most of this transcript is lost during RNA extraction, and only a small amount was degraded in the PAXgene tube (Fig. 7e, j).

*ZNF680* was the only gene examined where expression was higher in PAXgene than in Tempus RNA samples (Fig. 7i). *ZNF680* was also higher in PAXgene vs. Tempus lysate samples, suggesting that this transcript may not be as well preserved in Tempus collection tubes. Expression was only slightly higher in Tempus blood lysates compared to Tempus RNA samples, indicating only slight loss (<20%) of *ZNF680* during the Tempus RNA isolation procedure. Interestingly, a significant increase of *ZNF680* (~40%) was observed in PAXgene RNA vs. PAXgene lysate samples. The reason for this is unclear, but it is possible that loss of high expressing transcripts, such as ribosomal proteins (Fig. 3b), in the PAXgene RNA samples may result in the relative enrichment of other genes.

These data demonstrate that differences in gene expression seen in PAXgene vs. Tempus-processed samples are mainly due to loss of transcripts during the RNA extraction step using the PAXgene system. This indicates that the gene expression profile of Tempus-processed samples more accurately reflects the true gene expression profile, and that sample-processing dependent changes in gene expression could be minimized by measuring gene expression directly in the whole blood lysate.

## Discussion

We observed that approximately 5% of genes expressed in PBC are affected by sample processing, and that a significant number of these overlapped with genes that have previously been reported to change in PBC of at-risk AA+ first-degree relatives of T1D patients, and recent onset T1D patients compared to controls [3, 4]. Using samples from established T1D patients and healthy controls, we showed that combining PAXgene and Tempus-processed samples affected the gene expression profile and resulted in misleading changes in gene expression. We showed that differences in PBC gene expression can result from differences in transcript preservation in PAXgene or Tempus tubes, but were mainly due to loss or incomplete recovery of transcripts when RNA was extracted using the PAXgene system. Thus, the gene expression profiles of Tempus-processed RNA samples or minimally-processed whole blood lysates more accurately reflect the actual transcriptome than those of PAXgene-processed RNA.

This information is exceedingly important because gene expression profiling of peripheral blood in high-risk T1D-related individuals can potentially lead to the identification of diagnostic biomarkers of disease risk and progression – information that may provide insight into disease pathogenesis. Even using multi-center T1D consortia that have been established to collect biological samples from different cohorts of at-risk individuals, samples are frequently limited or collected from a particular demographic. Therefore, it is beneficial for researchers to combine data on samples from different sources.

Samples collected in the same type of collection tubes may also be processed differently between different consortia. For example, the TrialNet T1D samples used in this study were collected in Tempus tubes and processed using the KingFisher Purification system, an automated system that utilizes magnetic beads for RNA separation and purification. The Tempus Spin RNA isolation kit utilizes a spin column containing a silica membrane for RNA binding and elution. Because the spin columns are not optimal for retaining smaller transcripts, it is likely that these smaller transcripts are under-represented in samples extracted using the Tempus Spin RNA Isolation kit, and thus may have a different gene expression profile compared to samples processed using the KingFisher system. Our findings, however, do show that the majority of genes that are affected by processing using the Tempus Spin RNA isolation kit compared to the PAXgene blood RNA isolation kit do not significantly differ between KingFisher-processed TN-T1D samples and the Tempus system-processed control samples (Figs. 5b and 6; Set 2 genes).

Previous studies using a small number of samples (*n* = 3), or on a small number of genes, suggested that the choice of collection tube influenced gene expression [11, 13, 20, 21]. In this study, we demonstrate using 9 matched samples, that the collection tube and RNA extraction step affected the expression of ~900 genes. Some differences resulted from incomplete preservation of transcripts by the PAXgene or Tempus RNA-stabilizing reagent in the collection tubes, but the majority of differences are due to loss of certain mRNA transcripts when RNA was extracted using the PAXgene Blood RNA Kit.

RNA yield and quality have been reported to differ between PAXgene and Tempus samples [5, 11, 13, 16]. However, we and others observed little difference between the two systems (Table 1, [12, 14, 20]). These differences should not significantly alter relative gene expression levels, assuming genes of interest and housekeeping genes are uniformly affected. We found that the majority of genes (95%) were not significantly changed by sample processing. This is consistent with published studies showing little difference in the expression of select genes (*MMP9, ARG1*) in PAXgene vs. Tempus-processed RNA samples [20]. We showed that the expression of most housekeeping genes was not significantly affected by sample processing (Fig. 3c). However, *RNA18S5*, *B2M* and *TFRC* did fluctuate by ~1.5 to 4-fold in Tempus vs. PAXgene samples, and could significantly influence gene expression in PAXgene vs. Tempus samples if used for data normalization. Previous work has shown that the expression of *SIGLEC-7* measured in PAXgene and Tempus samples differs depending on the housekeeping gene used for normalization [11].

Nine hundred one genes were significantly affected by sample processing using the PAXgene vs. Tempus system. Most were downregulated in PAXgene samples and were highly enriched in ribosomal protein and lymphocyte-signature genes (Fig. 3, [17]). Surprisingly, the affected genes were also enriched for genes that are changed in the blood of at-risk AA+ first-degree relatives of T1D patients, recent onset [3, 4] or established T1D patients (Figs. 3d and 5). A number of ribosomal and oxidative phosphorylation pathway genes that are affected also overlapped with genes that are changed in the PBC of obese individuals and those suffering from metabolic syndrome [22, 23].

A similar study by Nikula et al. comparing PBC gene expression in PAXgene vs. Tempus samples showed differences in the expression of 443 genes [13]. 264 of these are present in our array, and 50 overlapped with our list of differentially expressed genes (see Additional file 4). Interestingly, Nikula et al. showed an enrichment of genes involved in RNA processing and immune cell function among the changed genes, and demonstrated that the gene expression profile of PBMCs is more similar to that of blood collected in Tempus than PAXgene tubes [13]. This supports our observation that lymphocyte-signature genes are lower in PAXgene samples. Another study showed that immune-related genes were upregulated in PHA-stimulated vs. control samples collected in Tempus, but not PAXgene tubes [15]. These include *IFNG* and *IL4*, which are known to respond to PHA stimulation. We found that *IFNG* and *IL4* were significantly lower in RNA prepared using the PAXgene system (see Additional file 5). Thus, PHA-induced changes seen in PAXgene-processed samples may not be as robust as those seen in Tempus-processed RNA samples.

By comparing gene expression in matching samples of whole blood lysate and RNA extracted from Tempus and PAXgene collection tubes, we asked if differences in gene expression were due to degradation of transcripts in the collection tube or loss of transcripts during the RNA extraction procedure. NanoString arrays were used to directly quantify mRNA transcripts in whole blood lysates and RNA samples without an amplification step, which may artificially distort gene expression levels [24, 25]. NanoString data were highly comparable to microarray and QPCR data and resulted in the least amount of variation among samples (Fig. 4). By comparing gene expression in whole blood lysates and total RNA of samples prepared in Tempus and PAXgene tubes, we showed that the Tempus Spin RNA Isolation Kit had little effect on gene expression. However, RNA extraction using the PAXgene Blood RNA Kit resulted in lower expression of multiple lymphocyte-signature genes and genes that reportedly are changed in the PBC of at-risk or recent-onset T1D patients (Figs. 3 and 7a-f [3, 4]). Previous studies have shown that a number of these genes including *COX6C, COX7B,* and *RPS24* (Fig. 2) were also reduced in samples prepared using the PAXgene system vs. the QIAamp system, which lyses cells in a buffer similar to the reagent contained in Tempus tubes [26]. Several genes (*KIF20B, KIAA1841* and *ZNF680*) were found to be better preserved in either PAXgene or Tempus tubes (Fig. 7g-i). Since PAXgene and Tempus tubes are highly acidic and basic, respectively, it is possible that certain gene transcripts are more stable in acidic or alkaline conditions [27].

It is unclear why RNA extraction of PAXgene samples leads to differences in gene expression compared to Tempus samples. Both RNA extraction kits utilize a silica membrane-based column for RNA binding and purification. However, optimal binding requires high pH and salt conditions [28]. It is possible that residual tartaric acid present in the PAXgene samples affect RNA binding to the column. This may explain the lower RNA yields previously observed in PAXgene vs. Tempus samples, but it is unclear why certain gene transcripts were more affected than others [5, 11, 13, 16].

## Conclusion

Our findings show that ~5% of genes expressed in PBCs are significantly affected by sample processing using the Tempus versus the PAXgene system. If the specific genes of interest are known, the most accurate method of quantification is direct measurement in blood lysates using NanoString arrays. This procedure uses the same volume of blood (collected in either Tempus or PAXgene tubes), does not require RNA extraction or gene amplification and results in the most consistent gene expression profiles between samples collected in PAXgene and Tempus tubes. For microarray, RNAseq, or QPCR experiments, where purified RNA is required, samples collected and processed using the Tempus system are recommended, especially for studies of immune function and T1D. Incomplete recovery or degradation of many more gene transcripts were observed using the PAXgene than the Tempus system, and the genes affected were highly enriched in genes that are reported to be dysregulated in the PBC of at-risk, recent onset, and established T1D patients [3, 4]. These differences need to be considered in any study, including meta-analysis studies [29] that combine expression data from PAXgene and Tempus samples.

## Additional files

Additional file 1: Differentially expressed genes in peripheral blood of female vs. male subjects. List of genes that are differentially expressed in the peripheral blood of female vs. male subjects, gene descriptions, and fold-change data. (DOCX 89 kb)

Additional file 2: Genes detected only using the PAXgene or the Tempus system. List of genes that are detected in the majority of samples collected in one type of tube (PAXgene or Tempus), but not detected in any samples collected with the other type of tube. Microarray probe IDs and gene descriptions are also included. (DOCX 84 kb)

Additional file 3: Correction Factor to convert expression in PAXgene-processed samples to expression in Tempus-processed samples. Correction factor (based on NanoString gene expression data) to convert expression of *COMMD6, COX6C, COX7B, LSM3, RPS24*, and *SUB1* measured in PAXgene-processed RNA samples to expression measured in Tempus-processed RNA samples. (DOCX 46 kb)

Additional file 4: Comparison of “collection-tube dependent” genes. Genes that are significantly changed by at least 2-fold in samples processed using the PAXgene versus Tempus system in our study, and in a previous study published by Nikula et al. (DOCX 101 kb)

Additional file 5: Genes changed by at least 2-fold in samples processed using the PAXgene versus Tempus system. The list of 901 genes that are significantly changed by at least 2-fold in samples processed using the PAXgene versus Tempus system. The fold change and microarray probe ID are included. (PDF 127 kb)
