# Supplementary material for: Impact of blood collection and processing on peripheral blood gene expression profiling in type 1 diabetes
Source: BMC Genomics. 2017 Aug 18;18:636. doi: 10.1186/s12864-017-3949-2 (PMC5563008; doi:10.1186/s12864-017-3949-2)
Supplement: Supplementary file 1 — Differentially expressed genes in peripheral blood of female vs. male subjects. List of genes that are differentially expressed in the peripheral blood of female vs. male subjects, gene descriptions, and fold-change data. (DOCX 89 kb) [file 12864_2017_3949_MOESM1_ESM.docx]

**Additional File 1**

**Differentially expressed genes in peripheral blood of female vs. male subjects**

| **Fold change*** | **Gene** | **Description** |
| --- | --- | --- |
| -2774.1 | *RPS4Y1* | ribosomal protein S4, Y-linked 1 |
| -1622.3 | *RPS4Y2* | ribosomal protein S4, Y-linked 2 |
| -460.1 | *DDX3Y* | DEAD (Asp-Glu-Ala-Asp) box helicase 3, Y-linked |
| -374.1 | *EIF1AY* | eukaryotic translation initiation factor 1A, Y-linked |
| -204.7 | *USP9Y* | ubiquitin specific peptidase 9, Y-linked |
| -78.9 | *UTY* | ubiquitously transcribed tetratricopeptide repeat containing, Y-linked |
| -33.6 | *TTTY14* | testis-specific transcript, Y-linked 14 |
| -27.5 | *ZFY* | zinc finger protein, Y-linked |
| -12.6 | *KDM5D* | lysine (K)-specific demethylase 5D |
| -11.2 | *TXLNGY* | taxilin gamma pseudogene, Y-linked |
| -10.7 | *TTTY15* | testis-specific transcript, Y-linked 15 |
| -7.3 | *FAM224A* | family with sequence similarity 224, member A, long non-coding RNA |
| -5.6 | *KCNT1* | potassium channel, subfamily T, member 1 |
| -4.1 | *DDX43* | DEAD (Asp-Glu-Ala-Asp) box polypeptide 43 |
| -3.3 | *TMSB4Y* | thymosin beta 4, Y-linked |
| -2.5 | *PRKY* | protein kinase, Y-linked, pseudogene |
| -2.0 | *ERICH1* | glutamate-rich 1 |
| 11.5 | *TSIX* | TSIX transcript, XIST antisense RNA |
| 63.2 | *lnc-CHIC1-2* | LNCipedia lincRNA |
| 148.4 | *XIST* | X inactive specific transcript |

*Fold change in female vs. male subjects (Moderated T-test with Benjamini Hochberg correction (P<0.05)
