# Supplementary material for: Impact of blood collection and processing on peripheral blood gene expression profiling in type 1 diabetes
Source: BMC Genomics. 2017 Aug 18;18:636. doi: 10.1186/s12864-017-3949-2 (PMC5563008; doi:10.1186/s12864-017-3949-2)
Supplement: Supplementary file 2 — Genes detected only using the PAXgene or the Tempus system. List of genes that are detected in the majority of samples collected in one type of tube (PAXgene or Tempus), but not detected in any samples collected with the other type of tube. Microarray probe IDs and gene descriptions are also included. (DOCX 84 kb) [file 12864_2017_3949_MOESM2_ESM.docx]

**Additional File 2**

**Genes detected only the PAXgene or the Tempus system**

| **Probe ID** | **Detected out of 9** | | **Gene** | **Description** |
| --- | --- | --- | --- | --- |
|  | **Tempus** | **PAXgene** |  |  |
| A_23_P122976 | 6 | 0 | *GNAI1* | guanine nucleotide binding protein, alpha inhibiting activity polypeptide 1 |
| A_23_P36641 | 5 | 0 | *AICDA* | activation-induced cytidine deaminase |
| A_33_P3342938 | 5 | 0 | *MEIS1* | Meis homeobox 1 |
| A_23_P43415 | 5 | 0 | *HSD17B3* | hydroxysteroid (17-beta) dehydrogenase 3 |
| A_23_P41344 | 5 | 0 | *EREG* | epiregulin |
| A_23_P18684 | 5 | 0 | *CLGN* | calmegin |
| A_23_P91081 | 7 | 0 | *EPCAM* | epithelial cell adhesion molecule |
| A_24_P12059 | 6 | 0 | *GYPA* | glycophorin A (MNS blood group) |
| A_24_P401615 | 0 | 5 | *PRAMEF10* | PRAME family member 10 |
| A_33_P3245517 | 0 | 5 | *LOC441666* | zinc finger protein 91 pseudogene |
| A_33_P3342153 | 0 | 5 | *USP17L8* | ubiquitin specific peptidase 17-like family member 8 |
| A_32_P16007 | 0 | 5 | *POTEB3* | POTE ankyrin domain family, member B3 |
| A_33_P3605352 | 0 | 5 | *WWTR1* | WW domain containing transcription regulator 1 |
| A_33_P3241378 | 0 | 5 | *POM121L8P* | POM121 transmembrane nucleoporin-like 8 pseudogene |
| A_24_P634768 | 0 | 5 | *FLJ22763* | uncharacterized LOC401081 |
| A_23_P428373 | 0 | 7 | *REXO1L1P* | REX1 RNA exonuclease 1 homolog-like 1 pseudogene |
| A_33_P3286980 | 0 | 5 | *USP17L1* | ubiquitin specific peptidase 17-like family member 1 |
