# Supplementary material for: Impact of blood collection and processing on peripheral blood gene expression profiling in type 1 diabetes
Source: BMC Genomics. 2017 Aug 18;18:636. doi: 10.1186/s12864-017-3949-2 (PMC5563008; doi:10.1186/s12864-017-3949-2)
Supplement: Supplementary file 3 — Correction Factor to convert expression in PAXgene-processed samples to expression in Tempus-processed samples. Correction factor (based on NanoString gene expression data) to convert expression of COMMD6, COX6C, COX7B, LSM3, RPS24, and SUB1 measured in PAXgene-processed RNA samples to expression measured in Tempus-processed RNA samples. (DOCX 46 kb) [file 12864_2017_3949_MOESM3_ESM.docx]

**Additional File 3**

**Correction Factor to convert expression in PAXgene-processed samples to expression in Tempus-processed samples**

|  | **Correction Factor*** | |
| --- | --- | --- |
| **Gene** | **Controls 1-9** | **Samples 1-11** |
| *COMMD6* | 5.4 ± 0.8 | 4.8 ± 0.8 |
| *COX6C* | 3.3 ± 0.4 | 3.4 ± 0.5 |
| *COX7B* | 2.7 ± 0.3 | 2.6 ± 0.3 |
| *LSM3* | 3.5 ± 0.4 | 2.7 ± 0.3 |
| *RPS24* | 8.8 ± 1.4 | 7.4 ± 1.1 |
| *SUB1* | 3.4 ± 0.5 | 2.8 ± 0.4 |
