# Supplementary material for: Impact of blood collection and processing on peripheral blood gene expression profiling in type 1 diabetes
Source: BMC Genomics. 2017 Aug 18;18:636. doi: 10.1186/s12864-017-3949-2 (PMC5563008; doi:10.1186/s12864-017-3949-2)
Supplement: Supplementary file 4 — Comparison of “collection-tube dependent” genes. Genes that are significantly changed by at least 2-fold in samples processed using the PAXgene versus Tempus system in our study, and in a previous study published by Nikula et al. (DOCX 101 kb) [file 12864_2017_3949_MOESM4_ESM.docx]

**Additional File 4**

**Table: Comparison of “collection-tube dependent” genes**

| **Gene** | **Fold change: PAXgene vs. Tempus** | | **Definition** |
| --- | --- | --- | --- |
|  | **Current study** | **Nikula et al.** |  |
| *CTSZ* | 2.5 | 3.1 | cathepsin Z |
| *PSMD5* | 2.3 | 2.4 | proteasome 26S subunit, non-ATPase, 5 |
| *AGER* | -2.5 | -4.6 | advanced glycosylation end product-specific receptor |
| *BIRC2* | -2.4 | -3.8 | baculoviral IAP repeat-containing 2 |
| *BNIP3L* | -4.7 | -2.8 | BCL2/adenovirus E1B 19kDa interacting protein 3-like |
| *CCAR1* | -3.0 | -165.4 | cell division cycle and apoptosis regulator 1 |
| *CLK1* | -2.5 | -2.5 | CDC-like kinase 1 |
| *CLU* | -2.8 | -6.4 | clusterin |
| *COX6C* | -3.4 | -2.2 | cytochrome c oxidase subunit Vic |
| *COX7C* | -2.5 | -2.8 | cytochrome c oxidase subunit VIIc, |
| *F13A1* | -2.1 | -2.8 | coagulation factor XIII, A1 polypeptide |
| *FKBP3* | -2.1 | -11.7 | FK506 binding protein 3, 25kDa |
| *GOLGA4* | -2.7 | -5.2 | golgi autoantigen, golgin subfamily a, 4 |
| *HAT1* | -3.0 | -26.0 | histone acetyltransferase 1 |
| *HMGB2* | -2.9 | -2.2 | high-mobility group box 2 |
| *MTPN* | -2.0 | -7.4 | myotrophin |
| *NUCB2* | -2.6 | -20.4 | nucleobindin 2 |
| *PFDN5* | -2.6 | -4.0 | prefoldin 5, transcript variant 3 |
| *PPBP* | -3.5 | -5.8 | pro-platelet basic protein |
| *PSMA4* | -2.3 | -2.4 | proteasome (prosome, macropain) subunit, alpha type, 4 |
| *RGS2* | -2.3 | -2.4 | regulator of G-protein signalling 2, 24kDa |
| *RPS24* | -5.4 | -8.6 | ribosomal protein S24 |
