# Supplementary material for: Impact of blood collection and processing on peripheral blood gene expression profiling in type 1 diabetes
Source: BMC Genomics. 2017 Aug 18;18:636. doi: 10.1186/s12864-017-3949-2 (PMC5563008; doi:10.1186/s12864-017-3949-2)
Supplement: Supplementary file 5 — Genes changed by at least 2-fold in samples processed using the PAXgene versus Tempus system. The list of 901 genes that are significantly changed by at least 2-fold in samples processed using the PAXgene versus Tempus system. The fold change and microarray probe ID are included. (PDF 127 kb) [file 12864_2017_3949_MOESM5_ESM.pdf]

Genes changed by at least 2-fold in PAXgene vs Tempus-processed tubes (P<0.05)

Probe ID	FC (PAX vs Tempus)	Gene
A_19_P00321068	2.01	MIAT
A_33_P3401058	2.01	lnc-KIAA0825-1
A_33_P3242383	2.01	AFF1
A_32_P39003	2.02	LOC100133050
A_33_P3300346	2.02	DCUN1D2
A_33_P3215838	2.02	KLK3
A_33_P3379046	2.02	LOC729770
A_33_P3394517	2.02	SMG1
A_33_P3307965	2.03	AADACL3
A_33_P3788772	2.03	LOC200609
A_23_P9018	2.03	DEFB104B
A_33_P3336240	2.03	DEFB109P1
A_33_P3233010	2.04	KIR3DL2
A_23_P301247	2.04	HIST2H2AC
A_24_P142743	2.04	CNN2
A_19_P00317610	2.04	LOC284930
A_23_P30813	2.05	HIST1H4K
A_33_P3214422	2.06	ANP32A
A_33_P3218301	2.06	GPAT2
A_24_P238143	2.06	LRRC37A2
A_23_P29985	2.06	TRMT44
A_33_P3236734	2.07	CLEC17A
A_24_P522864	2.07	MGC12916
A_33_P3380837	2.08	AMZ1
A_23_P161918	2.08	CCDC86
A_33_P3368188	2.08	SEPT9
A_23_P381461	2.08	LRRC45
A_33_P3314471	2.08	IMMT
A_33_P3344991	2.08	TBC1D3L
A_33_P3306854	2.08	LOC400553
A_33_P3321533	2.08	TMEM178B
A_33_P3423700	2.08	LOC100131829
A_24_P117942	2.08	TOMM20L
A_33_P3265956	2.08	CBFA2T2
A_32_P179837	2.08	RNF151
A_24_P365753	2.09	NKAPP1
A_24_P136094	2.10	CCDC88C
A_33_P3215317	2.10	PRKACB
A_33_P3343981	2.10	AATK
A_33_P3373892	2.10	NAA35
A_33_P3239914	2.10	LOC100130741
A_33_P3389178	2.10	RAPGEF3
A_33_P3273969	2.10	ZC3H11A
A_19_P00808348	2.11	LOC101927151
A_23_P14649	2.11	ANP32A-IT1
A_33_P3362326	2.11	FHL2
A_19_P00808297	2.11	PLEKHA3
A_19_P00322299	2.11	ZSWIM6
A_33_P3332885	2.11	BTN2A1
A_33_P3812815	2.12	PKD1
A_33_P3325195	2.12	IGSF9B
A_33_P3333992	2.12	LOC100132495
A_33_P3218975	2.13	ENTPD1
A_33_P3285799	2.13	AK9
A_33_P3340294	2.13	RBM33
A_19_P00320721	2.13	MDM4
A_33_P3214310	2.13	FOXP1
A_33_P3214785	2.14	LEPROT
A_33_P3267118	2.14	EIF4G3
A_33_P3335183	2.14	LRRC37A2
A_33_P3214432	2.14	ZC3HAV1L
A_23_P137504	2.14	ZBTB37
A_33_P3358158	2.15	CRYBB2P1
A_33_P3760125	2.15	LOC102725353
A_33_P3365878	2.15	BMP8B
A_33_P3398917	2.16	C15orf59
A_33_P3255499	2.16	SLC25A51
A_19_P00802018	2.16	lnc-GUSB-5
A_19_P00810156	2.16	KIFC1
A_24_P84822	2.17	GUSBP1
A_23_P26173	2.17	TMED3
A_33_P3295650	2.17	APBA1
A_33_P3365586	2.18	VAPB
A_33_P3649472	2.19	LOC283856
A_23_P102364	2.19	NGEF

Probe ID	FC (PAX vs Tempus)	Gene
A_32_P52119	2.19	LOC101928673
A_33_P3419733	2.19	DNAJC5
A_33_P3319113	2.19	PDPR
A_19_P00317978	2.20	ZNF207
A_32_P703	2.20	LOC646626
A_33_P3395274	2.22	ZADH2
A_33_P3323760	2.22	LOC153684
A_33_P3274622	2.23	LINC01347
A_33_P3237266	2.23	IDS
A_33_P3292179	2.23	ABCA9
A_33_P3267612	2.23	MIR143HG
A_24_P701776	2.23	ARHGEF35
A_23_P136573	2.23	ST3GAL5
A_19_P00318280	2.23	LOC100506860
A_19_P00319130	2.23	lnc-GPR20-4
A_33_P3349395	2.24	CECR2
A_33_P3322415	2.24	MARK4
A_33_P3224495	2.25	KCNQ5-IT1
A_33_P3395952	2.25	COL20A1
A_32_P88415	2.25	MYOZ3
A_23_P146134	2.25	DUSP26
A_33_P3330826	2.26	LOC100132077
A_19_P00321266	2.26	LINC00630
A_33_P3702281	2.27	LOC284014
A_23_P107775	2.27	TMEM190
A_33_P3305243	2.27	PSMD5
A_23_P318396	2.27	CELF1
A_33_P3388983	2.28	C5orf56
A_33_P3252228	2.28	NRSN1
A_23_P143650	2.29	DUSP18
A_23_P134729	2.29	RBM12B-AS1
A_23_P44724	2.29	CSRP2
A_33_P3802146	2.30	LINC00504
A_33_P3307980	2.30	lnc-FAM133B-1
A_19_P00317897	2.30	LOC101928673
A_33_P3216517	2.30	NEK6
A_33_P3247753	2.31	TSIX
A_32_P902957	2.31	ARL11
A_33_P3302676	2.31	TLE4
A_23_P30805	2.31	HIST1H4J
A_33_P3306048	2.31	DPRXP4
A_19_P00803086	2.32	lnc-RPRML-3
A_33_P3359308	2.32	KLHL29
A_19_P00813482	2.33	CRHR1-IT1
A_24_P927716	2.33	SLED1
A_23_P27133	2.33	KRT15
A_33_P3256510	2.33	KCNK12
A_33_P3231695	2.33	ZNF667
A_33_P3299160	2.34	LINC00544
A_19_P00809599	2.34	RRM1
A_33_P3275959	2.34	SLC25A16
A_24_P298027	2.35	AXIN2
A_19_P00801731	2.35	LOC102725511
A_33_P3371752	2.36	EPS15
A_24_P218006	2.36	DNAJC30
A_33_P3409245	2.36	LOC401286
A_23_P59138	2.36	POU5F1
A_19_P00319177	2.37	LOC401320
A_33_P3287862	2.37	LOC101060524
A_24_P65941	2.38	RUNX1-IT1
A_33_P3452003	2.39	LOC143286
A_33_P3212799	2.39	C22orf34
A_23_P132358	2.39	MCAT
A_23_P59988	2.39	SLC35G5
A_33_P3228739	2.39	LRRC3C
A_33_P3372705	2.39	SRGAP2B
A_33_P3831730	2.40	lnc-NDUFA4-2
A_19_P00317082	2.40	HCG18
A_23_P214821	2.40	EDN1
A_33_P3385567	2.40	ARNTL
A_33_P3411477	2.41	NCCRP1
A_23_P22614	2.41	SEPT6
A_19_P00325018	2.42	SAAL1
A_33_P3711318	2.42	LOC257152
A_33_P3381259	2.42	lnc-DNAI1-1

Probe ID	FC (PAX vs Tempus)	Gene
A_33_P3404189	2.42	MROH1
A_33_P3254695	2.42	SNORA73B
A_33_P3380405	2.42	CYTH1
A_33_P3281613	2.43	KCNT1
A_24_P159648	2.44	BAIAP2
A_23_P40240	2.45	CTSZ
A_33_P3315733	2.45	TRAPPC10
A_33_P3342111	2.45	ZNF169
A_33_P3277714	2.46	BACH2
A_33_P3409854	2.46	LHX1
A_33_P3410279	2.47	DOCK9
A_33_P3233649	2.47	NADK2
A_19_P00315551	2.47	LOC400043
A_33_P3372526	2.47	MDH1B
A_33_P3416588	2.47	RIT2
A_19_P00806753	2.47	LOC401320
A_33_P3278826	2.48	LTK
A_33_P3406961	2.49	XLOC_l2_013467
A_33_P3304824	2.49	LINC00618
A_23_P205575	2.49	GPR135
A_19_P00315716	2.51	SNORA71A
A_23_P348121	2.52	FOSL2
A_33_P3422429	2.52	INADL
A_19_P00801304	2.52	LOC102725134
A_23_P22352	2.52	FRMD4A
A_33_P3549874	2.52	LOC255177
A_33_P3464855	2.54	DKFZP586B0319
A_33_P3212665	2.56	HTATSF1P2
A_33_P3372368	2.56	KRT8
A_33_P3268564	2.56	NCK2
A_23_P70359	2.59	AGPAT4-IT1
A_23_P213857	2.59	C7
A_33_P3308223	2.60	PDE4DIP
A_33_P3406828	2.63	MAFIP
A_24_P693321	2.63	LOC100190986
A_33_P3356577	2.64	SIRPB1
A_33_P3242069	2.64	LOC729159
A_33_P3220390	2.64	WTAP
A_19_P00813124	2.65	XLOC_l2_010511
A_32_P212802	2.66	LOC101930277
A_19_P00316286	2.66	LOC401320
A_33_P3265415	2.67	XLOC_l2_013410
A_33_P3230017	2.68	AURKAPS1
A_23_P66827	2.68	FAM106A
A_24_P39639	2.68	EXOSC6
A_19_P00317023	2.68	lnc-MYC-2
A_33_P3227217	2.69	SNORA81
A_33_P3272399	2.69	LOC645427
A_33_P3301689	2.71	ZNF326
A_24_P941866	2.71	C5orf56
A_33_P3375451	2.73	NYNRIN
A_32_P151544	2.73	KRT18
A_23_P377411	2.73	LOC100133130
A_33_P3421365	2.74	ZNF169
A_33_P3303176	2.74	MRGPRG
A_33_P3257513	2.74	FAT3
A_33_P3294951	2.74	TAS2R30
A_33_P3326020	2.75	KANK3
A_23_P326142	2.76	SND1-IT1
A_33_P3231319	2.77	XLOC_l2_013192
A_23_P105495	2.79	TAS2R14
A_33_P3384667	2.80	DCAF5
A_33_P3389599	2.80	ANKRD20A11P
A_33_P3244728	2.81	LRP2
A_33_P3527721	2.82	LOC284219
A_33_P3210583	2.82	AAK1
A_24_P93206	2.82	TMEM179
A_33_P3404641	2.83	PRR34-AS1
A_23_P57733	2.83	DRD3
A_33_P3336592	2.85	KLF13
A_24_P728006	2.85	XLOC_l2_011874
A_23_P207680	2.86	ARL17B
A_33_P3382595	2.86	RN7SK
A_33_P3397795	2.86	PCNXL4
A_33_P3382276	2.89	ST6GAL1
A_33_P3419360	2.90	LINC00202-2
A_19_P00319456	2.90	ZNF83
A_33_P3709317	2.93	SNORA28
A_33_P3415744	2.95	ANKRD36B
A_23_P371076	2.96	KLF12
A_24_P106542	2.97	RSPO3
A_33_P3219459	2.97	TMEM240
A_33_P3314356	2.97	PPM1K
A_33_P3275315	2.97	CELF2-AS1
A_23_P387000	3.02	XKR6
A_33_P3365087	3.02	PRR20B
A_19_P00319448	3.03	ZNF83
A_33_P3842770	3.04	LOC101928669
A_33_P3366073	3.05	EBPL
A_24_P312325	3.05	nc-AF131215.3.1-1
A_19_P00321866	3.06	C1orf132
A_33_P3263379	3.06	SNORD17
A_33_P3263851	3.07	LOC400863
A_33_P3518572	3.09	LINC00941
A_33_P3313825	3.10	TGFBR2
A_33_P3222367	3.14	SNORA74B
A_33_P3363120	3.14	SCARNA10
A_33_P3244117	3.14	STARD9
A_33_P3658861	3.15	SNORA26
A_32_P54274	3.16	DRD5
A_19_P00319422	3.17	KIAA1671
A_23_P433218	3.21	OR7E91P
A_33_P3318946	3.22	HAPLN2
A_24_P356	3.24	AAK1
A_23_P113777	3.26	ITGBL1
A_33_P3241884	3.26	SDC3
A_33_P3289034	3.28	PKD2L1
A_23_P74114	3.34	ZNF713
A_33_P3403643	3.35	LOC101059906
A_33_P3267263	3.37	RNVU1-18
A_19_P00804049	3.40	LOC401320
A_23_P130642	3.42	GBP6
A_33_P3236441	3.43	LDHAL6A
A_33_P3870056	3.43	LOC283335
A_33_P3499102	3.43	RBM10
A_33_P3317623	3.45	LOC284379
A_33_P3383071	3.51	LOC100128437
A_32_P120604	3.55	SNX29
A_33_P3325558	3.58	RABGAP1L
A_33_P3710442	3.65	FLJ11710
A_33_P3436646	3.67	LOC151657
A_33_P3355407	3.68	SNORA73B
A_33_P3209254	3.69	RPS16P5
A_33_P3665553	3.72	LOC100233156
A_24_P164505	3.75	FAM106CP
A_33_P3332348	3.88	RN7SL1
A_23_P156809	3.88	METTL21A
A_33_P3390107	3.89	RNA18S5
A_32_P846696	4.07	LINC00965
A_33_P3316052	4.07	CA5B
A_19_P00322228	4.12	LOC102725166
A_33_P3236319	4.15	lnc-CBWD5-2
A_19_P00320047	4.15	nc-AC069257.9.1-4
A_23_P106682	4.19	EMP2
A_33_P3503937	4.20	LOC284581
A_33_P3363082	4.20	SCARNA5
A_33_P3316505	4.24	SNORA73A
A_33_P3299921	4.29	lnc-CBWD5-2
A_33_P3414964	4.52	PTPRS
A_24_P933011	4.54	TMEM200C
A_23_P408930	4.66	OR7E5P
A_33_P3396444	4.68	PTGR1
A_33_P3279708	5.00	RNU2-1
A_33_P3276142	5.08	DNM1P46
A_33_P3378384	5.15	DNM1P46
A_33_P3249076	5.18	HOGA1
A_19_P00800206	5.62	LOC400958
A_33_P3405916	7.61	LINC00273
A_33_P3389394	9.14	RPPH1

Probe ID	FC (PAX vs Tempus)	Gene
A_33_P3306153	-21.08	KIAA1841
A_33_P3215239	-17.15	KIF20B
A_23_P31671	-7.59	UQCRB
A_19_P00811288	-7.43	UQCRBP1
A_23_P43412	-6.92	HEMGN
A_24_P414658	-6.81	HIST1H2AG
A_24_P387609	-6.77	ISCA1
A_23_P7732	-6.74	CETN3
A_23_P213706	-6.72	IL4
A_33_P3288159	-6.65	ASPM
A_23_P145761	-6.63	ARL4A
A_23_P166023	-6.51	PFDN4
A_33_P3233916	-6.28	SOX6
A_24_P237613	-6.24	USP12
A_33_P3385842	-6.09	CCDC7
A_33_P3341429	-5.98	NEXN
A_23_P43810	-5.94	LTBP1
A_23_P63158	-5.75	SPTA1
A_23_P97141	-5.67	RGS1
A_24_P188878	-5.63	RPL34
A_32_P167396	-5.56	MED14OS
A_32_P102062	-5.50	FOXO3
A_23_P65768	-5.43	RSL24D1
A_23_P149476	-5.42	EFCAB2
A_24_P943301	-5.41	PEAR1
A_23_P1206	-5.38	RPS24
A_23_P217428	-5.33	ARHGAP6
A_24_P921366	-5.31	CALD1
A_33_P3392921	-5.23	CTTN
A_23_P2414	-5.19	SPX
A_23_P57277	-5.07	MAP3K7CL
A_23_P136909	-5.00	HMGN5
A_23_P397480	-5.00	ABCC13
A_33_P3230269	-4.97	GRHL1
A_23_P314805	-4.93	TMEM56
A_23_P73239	-4.74	NCKAP1
A_33_P3304655	-4.70	LTBP1
A_23_P125705	-4.68	NAP1L2
A_33_P3396951	-4.67	BNIP3L
A_23_P312246	-4.64	CCDC82
A_23_P152002	-4.61	BCL2A1
A_24_P215240	-4.59	ENKUR
A_19_P00800264	-4.59	OTUD6B-AS1
A_24_P944588	-4.58	ZNF682
A_32_P12580	-4.57	SMAD2
A_23_P155989	-4.54	CENPK
A_23_P215751	-4.50	NDUFA5
A_23_P121564	-4.46	GUCY1B3
A_24_P944458	-4.45	INSIG2
A_24_P403303	-4.42	PHF20L1
A_33_P3413741	-4.42	OXTR
A_32_P145153	-4.39	RPL31
A_33_P3887888	-4.39	FLJ44511
A_23_P159650	-4.38	COX7B
A_23_P64173	-4.36	CARD16
A_32_P31182	-4.33	RPL7
A_23_P7221	-4.31	RPL34
A_33_P3413038	-4.31	PLXNB3
A_23_P331928	-4.29	CD109
A_24_P29001	-4.29	LSM3
A_23_P128991	-4.29	SLIRP
A_23_P155979	-4.24	EGF
A_24_P141736	-4.23	METAP2
A_24_P111242	-4.19	SVIP
A_24_P46334	-4.17	SCOC
A_32_P114215	-4.16	COMMD6
A_33_P3262485	-4.16	ZNF563
A_24_P393958	-4.16	DNAJB4
A_23_P73114	-4.15	PROS1
A_23_P151294	-4.14	IFNG
A_33_P3370226	-4.13	RPL7
A_23_P256572	-4.11	RHAG
A_33_P3248519	-4.08	SMC4
A_33_P3236071	-4.05	CLEC1B
A_33_P3414202	-4.04	MBNL3
A_23_P161399	-4.02	MXI1
A_24_P475349	-4.01	RAB6B
A_23_P154526	-4.01	GRB14
A_33_P3236065	-4.01	CLEC1B
A_23_P386964	-3.99	C4orf45
A_33_P3327165	-3.97	CCDC18
A_33_P3828101	-3.97	CCDC112
A_24_P362540	-3.96	ASAP2

Probe ID	FC (PAX vs Tempus)	Gene
A_24_P320328	-3.95	SUB1
A_33_P3303449	-3.93	LEPR
A_24_P100830	-3.93	AMN1
A_33_P3323136	-3.92	ENKUR
A_23_P252201	-3.88	EAF2
A_24_P192805	-3.87	CARD17
A_19_P00800114	-3.87	HIGD1A
A_24_P681011	-3.87	HIPK2
A_33_P3217230	-3.86	ZNF91
A_23_P87879	-3.84	CD69
A_24_P346126	-3.83	SERF1A
A_33_P3332955	-3.82	CLEC1B
A_32_P21384	-3.82	RPL17
A_23_P209032	-3.80	ZNF302
A_24_P56484	-3.79	BRMS1L
A_23_P206324	-3.78	HSDL1
A_23_P84821	-3.76	MRPL1
A_23_P33045	-3.74	RPL26
A_23_P217609	-3.72	RPL36A
A_23_P45087	-3.71	ZNF107
A_23_P14734	-3.70	RPS27L
A_32_P109683	-3.69	PAGE2B
A_24_P104407	-3.68	SYNM
A_33_P3280521	-3.68	MFAP3L
A_32_P55860	-3.65	SKA2
A_33_P3221443	-3.64	ZNF254
A_23_P410017	-3.64	TBCEL
A_23_P107724	-3.62	ZNF112
A_24_P390583	-3.62	USP31
A_23_P102235	-3.61	SNRPG
A_24_P156769	-3.60	MPL
A_23_P14708	-3.59	ZNF280D
A_33_P3317009	-3.58	SLC11A1
A_24_P601972	-3.58	C2orf74
A_24_P209171	-3.58	SH3BGRL2
A_23_P85726	-3.57	METTL18
A_33_P3235706	-3.55	ZCCHC11
A_32_P47870	-3.54	lnc-PDZD8-1
A_23_P60002	-3.54	EMC2
A_32_P73045	-3.54	CMAS
A_23_P160689	-3.53	LRIF1
A_23_P156620	-3.52	ZNF184
A_32_P18159	-3.51	LYRM7
A_33_P3344831	-3.50	TMEM45A
A_32_P930685	-3.50	ZNF876P
A_33_P3258274	-3.49	TFPI
A_23_P42664	-3.48	SHFM1
A_23_P411335	-3.46	SGOL2
A_23_P121596	-3.46	PPBP
A_33_P3406072	-3.46	FRMD3
A_23_P323751	-3.45	FAM83D
A_23_P359277	-3.44	ELOVL7
A_23_P52017	-3.44	ASPM
A_24_P535219	-3.43	PHF10
A_23_P8900	-3.42	COX6C
A_33_P3232552	-3.42	GUCY1B3
A_33_P3230189	-3.42	SLITRK6
A_24_P122337	-3.41	SYTL4
A_32_P203300	-3.41	EIF4E
A_23_P39542	-3.41	C2orf76
A_33_P3843415	-3.41	WDR11-AS1
A_24_P213783	-3.41	RPL31
A_23_P349343	-3.40	ALS2CR12
A_23_P50108	-3.38	NDC80
A_19_P00319990	-3.38	lnc-SNURF-1
A_23_P218068	-3.38	PLEKHA5
A_23_P431252	-3.37	KBTBD8
A_23_P321984	-3.37	CLECL1
A_23_P157449	-3.37	POLR2K
A_23_P8913	-3.36	CA2
A_24_P86993	-3.35	JAM3
A_23_P118516	-3.35	TVP23B
A_23_P203023	-3.34	RDX
A_23_P216766	-3.33	ISCA1
A_23_P156842	-3.33	EEF1E1
A_23_P113748	-3.32	ZNF385D
A_23_P143958	-3.32	RPL22L1
A_32_P199301	-3.32	TFDP1
A_33_P3320152	-3.31	SNRPE
A_23_P85682	-3.30	NFIA
A_24_P242132	-3.30	NRBP2
A_23_P125042	-3.30	ZNF222
A_24_P936145	-3.30	GNAO1

Probe ID	FC (PAX vs Tempus)	Gene
A_24_P115651	-3.29	ENKUR
A_23_P413303	-3.29	DKFZP434I0714
A_33_P3319625	-3.27	NFIA
A_32_P158746	-3.25	RPL17
A_32_P38467	-3.24	SNHG8
A_33_P3220207	-3.24	ARMC3
A_23_P115608	-3.24	ARHGAP21
A_32_P168349	-3.22	C6orf25
A_23_P335039	-3.20	ZNF721
A_23_P254978	-3.20	TATDN1
A_24_P941487	-3.19	ZNF761
A_23_P210274	-3.19	MOB4
A_24_P123408	-3.18	ABLIM3
A_33_P3303697	-3.18	CR2
A_23_P125771	-3.18	HCFC1
A_32_P69465	-3.17	MORN2
A_24_P407235	-3.16	CRY1
A_23_P133058	-3.16	MRFAP1L1
A_23_P149545	-3.15	HIST2H2BE
A_23_P124837	-3.15	LRP1
A_33_P3345414	-3.15	RPS4X
A_23_P83278	-3.15	CHMP5
A_33_P3542801	-3.15	LOC729291
A_23_P21316	-3.15	PRUNE
A_33_P3400477	-3.14	STIL
A_33_P3303372	-3.14	PARD3
A_23_P302005	-3.14	STON1
A_33_P3313785	-3.14	CCDC14
A_23_P164047	-3.13	MMD
A_33_P3390057	-3.13	TM4SF1
A_23_P202004	-3.12	PRTFDC1
A_23_P77145	-3.12	RAB11A
A_32_P155364	-3.12	RPL7
A_23_P48669	-3.12	CDKN3
A_23_P162918	-3.12	SERPINA3
A_32_P148672	-3.11	SNRPD1
A_33_P3393836	-3.11	NT5C3A
A_33_P3281191	-3.11	NID1
A_32_P76156	-3.10	RWDD4
A_33_P3775848	-3.10	CLIC2
A_33_P3779229	-3.09	GK5
A_33_P3403773	-3.09	ZNF569
A_23_P62953	-3.09	PBX1
A_23_P314115	-3.09	BMI1
A_24_P110780	-3.08	NEXN-AS1
A_24_P191790	-3.07	TMEM33
A_24_P336931	-3.07	ANKRD36
A_23_P144369	-3.07	NAP1L5
A_24_P247536	-3.07	FAM133B
A_23_P162596	-3.06	ACTR6
A_23_P126836	-3.05	TNFSF4
A_23_P115842	-3.04	CCAR1
A_33_P3390017	-3.03	C15orf54
A_23_P317347	-3.03	ESCO1
A_23_P933	-3.03	RWDD3
A_33_P3348288	-3.02	RHOBTB1
A_24_P253251	-3.02	SLC7A1
A_32_P196193	-3.02	PAQR9
A_23_P128930	-3.01	PSMC6
A_32_P9382	-3.01	MZT1
A_24_P132787	-3.01	RAB18
A_24_P196851	-3.01	TLN1
A_23_P42975	-3.00	PRKAR2B
A_24_P277367	-3.00	CXCL5
A_23_P207507	-3.00	ABCC3
A_23_P97457	-3.00	AIDA
A_24_P43876	-2.99	C9orf40
A_32_P49616	-2.99	EEF1B2
A_23_P339480	-2.99	HAT1
A_33_P3216714	-2.99	DNAJC6
A_23_P112452	-2.98	GGTA1P
A_24_P344961	-2.98	AMOT
A_23_P500998	-2.98	HOXA9
A_23_P56759	-2.98	KRCC1
A_23_P74349	-2.97	NUF2
A_23_P58396	-2.97	PDGFC
A_23_P417942	-2.96	FNBP1L
A_32_P34589	-2.95	RSRC1
A_23_P115366	-2.95	CMPK1
A_33_P3263902	-2.95	MXI1
A_23_P23748	-2.95	WDR47
A_23_P151133	-2.95	TSPAN9
A_24_P319736	-2.94	MEIS1

Probe ID	FC (PAX vs Tempus)	Gene
A_23_P23765	-2.94	ITGB3BP
A_24_P398130	-2.93	USP6NL
A_32_P186981	-2.93	RPL17
A_23_P347059	-2.93	MOB1B
A_32_P206949	-2.92	TMEM17
A_23_P134714	-2.92	HRSP12
A_32_P162250	-2.92	ARHGAP18
A_23_P17021	-2.92	SCRN3
A_23_P415706	-2.91	GPR133
A_24_P336705	-2.91	RABGGTB
A_23_P30307	-2.91	MED7
A_23_P155765	-2.90	HMGB2
A_23_P65278	-2.90	NBEA
A_33_P3343316	-2.89	SH3BGRL2
A_33_P3347330	-2.89	TOMM7
A_23_P210763	-2.89	JAG1
A_33_P3214293	-2.88	PPP3R1
A_32_P138396	-2.88	lnc-NUB1-1
A_24_P345209	-2.88	DYRK3
A_23_P89762	-2.87	PHLPP1
A_23_P380208	-2.87	VEPH1
A_23_P431319	-2.87	YIPF6
A_23_P34546	-2.87	SDCCAG8
A_23_P41942	-2.86	POLR3G
A_23_P135499	-2.86	CLIC4
A_23_P501080	-2.86	ZNF92
A_24_P65373	-2.86	ITGA2B
A_33_P3257943	-2.85	RPL26
A_23_P2129	-2.85	TMEM126B
A_32_P190416	-2.85	MAP7
A_24_P256692	-2.83	MIA3
A_23_P84070	-2.83	LARP7
A_24_P271049	-2.83	C18orf32
A_33_P3295578	-2.83	TCEAL8
A_23_P409541	-2.83	POLR1D
A_33_P3389133	-2.82	MINPP1
A_23_P137434	-2.82	RNF11
A_23_P110362	-2.81	LAMTOR3
A_23_P44257	-2.81	COMMD8
A_23_P67424	-2.81	ZNF461
A_33_P3234989	-2.80	IFT81
A_23_P120048	-2.80	BAZ2B
A_24_P252846	-2.80	C11orf74
A_23_P215913	-2.80	CLU
A_33_P3369520	-2.80	MAGI3
A_24_P182620	-2.80	CELSR2
A_33_P3257558	-2.79	CNTRL
A_33_P3227506	-2.79	BPTF
A_23_P5757	-2.79	TPRKB
A_23_P73702	-2.79	MED12
A_23_P351467	-2.78	CMAHP
A_33_P3363425	-2.78	FRMD3
A_23_P255714	-2.78	IFT74
A_33_P3278455	-2.78	ANKRD6
A_33_P3319026	-2.77	ZZZ3
A_23_P127579	-2.77	PTS
A_33_P3280030	-2.77	FRA10AC1
A_33_P3299872	-2.76	HINT3
A_24_P100551	-2.76	SH3RF1
A_23_P371266	-2.76	DNM3
A_23_P21734	-2.76	AK6
A_23_P411881	-2.76	PIBF1
A_23_P107644	-2.75	SNRPD1
A_24_P182122	-2.75	ND1
A_23_P254733	-2.74	CENPU
A_23_P302550	-2.74	RGS18
A_23_P102950	-2.74	RSPH1
A_33_P3360665	-2.73	ACVR1
A_23_P256391	-2.72	GOLGA4
A_24_P328872	-2.72	CCT5
A_33_P3256997	-2.72	MS4A4E
A_33_P3233947	-2.72	COX20
A_23_P133648	-2.71	FAM8A1
A_19_P00804527	-2.69	EIF2AK2
A_24_P148151	-2.69	TSNAX
A_23_P105535	-2.69	POC1B
A_24_P67946	-2.69	NUDT4
A_23_P250571	-2.69	DMXL1
A_23_P43946	-2.69	SARNP
A_33_P3332130	-2.69	PGRMC1
A_33_P3249434	-2.68	PLAA
A_23_P41159	-2.68	PPP4R2
A_33_P3409337	-2.67	CCDC176

Probe ID	FC (PAX vs Tempus)	Gene
A_23_P95594	-2.67	NAT1
A_23_P161686	-2.66	ARHGAP32
A_23_P94009	-2.66	LSM8
A_23_P21473	-2.66	CEP70
A_24_P915692	-2.66	PHLDA1
A_23_P170608	-2.66	TSPYL2
A_19_P00318652	-2.66	lnc-TCF24-2
A_23_P382835	-2.66	P2RY1
A_23_P89073	-2.66	ZNF23
A_23_P2041	-2.66	MICALCL
A_23_P19624	-2.65	BMP6
A_24_P917026	-2.65	NF1
A_33_P3239954	-2.65	USP14
A_23_P59358	-2.65	CEP57L1
A_23_P426663	-2.65	MITF
A_24_P282043	-2.65	ZNF28
A_23_P427114	-2.65	GORASP1
A_23_P140035	-2.64	WBP4
A_24_P167654	-2.64	SLC8A3
A_24_P28295	-2.64	RABGAP1L
A_23_P115011	-2.64	ADAMTSL4
A_23_P21785	-2.64	NSUN3
A_23_P361448	-2.64	SESN3
A_24_P16913	-2.64	ABCC4
A_23_P395075	-2.63	KDM3A
A_23_P71053	-2.63	MPP6
A_33_P3360942	-2.63	SCAMP1
A_24_P64167	-2.63	PTGS1
A_23_P357760	-2.62	ARSD
A_23_P205646	-2.62	MAP4K5
A_23_P431853	-2.62	ND2
A_24_P115529	-2.62	LOC51145
A_24_P114339	-2.61	RMND5A
A_33_P3401647	-2.61	PPP1R14A
A_23_P75038	-2.61	DCLRE1A
A_32_P148824	-2.61	C1orf27
A_24_P73669	-2.60	GSPT1
A_23_P56298	-2.60	ZNF430
A_24_P181120	-2.60	PFDN5
A_23_P161909	-2.60	MS4A3
A_23_P129577	-2.60	TIGD7
A_24_P371399	-2.60	C3orf58
A_33_P3240263	-2.59	POMP
A_24_P288754	-2.58	PIGA
A_33_P3405743	-2.58	CRYZ
A_23_P72503	-2.58	KLHL2
A_23_P26713	-2.58	RPL23
A_33_P3317073	-2.57	MOB1B
A_23_P303155	-2.57	TMEM87B
A_24_P228550	-2.57	TUBB1
A_33_P3413558	-2.57	CD226
A_19_P00813077	-2.57	FAM228B
A_23_P316501	-2.57	NKAIN2
A_23_P73835	-2.57	MOSPD1
A_32_P138617	-2.56	KIAA2018
A_23_P13364	-2.56	NUCB2
A_23_P141549	-2.55	RPS7
A_23_P2922	-2.55	MBIP
A_32_P75299	-2.55	TOMM5
A_23_P72387	-2.55	AFAP1
A_33_P3248992	-2.55	ACADSB
A_23_P144497	-2.55	RPS3A
A_33_P3272461	-2.54	METTL5
A_23_P16817	-2.54	CLK1
A_24_P295543	-2.54	BLOC1S2
A_33_P3367247	-2.54	CNTLN
A_23_P370830	-2.54	KLHL14
A_23_P164559	-2.52	CHAMP1
A_23_P104876	-2.52	SPA17
A_24_P941930	-2.51	EAF1
A_23_P122007	-2.51	C5orf30
A_24_P945059	-2.50	MYCT1
A_23_P344451	-2.50	HDGFRP3
A_24_P943106	-2.50	U2SURP
A_33_P3396008	-2.50	AGER
A_23_P253158	-2.50	EP400
A_23_P153037	-2.50	ZNF624
A_23_P46369	-2.49	RAB13
A_33_P3269110	-2.49	GUCY1A3
A_33_P3260605	-2.49	CTNNAL1
A_24_P115007	-2.48	ALDH5A1
A_32_P69166	-2.48	ANKRD42
A_24_P942630	-2.48	KDM6B

Probe ID	FC (PAX vs Tempus)	Gene
A_23_P233	-2.47	FMO5
A_33_P3367062	-2.47	SWT1
A_24_P36097	-2.47	RPAIN
A_33_P3333712	-2.47	SCYL2
A_23_P162734	-2.46	RNF6
A_33_P3244181	-2.46	HSBP1
A_33_P3413597	-2.46	NDUFB3
A_24_P171058	-2.46	TMEM64
A_23_P56380	-2.46	ZC3H15
A_23_P110811	-2.46	COX7C
A_19_P00811048	-2.45	ZEB1-AS1
A_24_P319923	-2.45	MYLK
A_24_P92952	-2.44	ARID1A
A_24_P115774	-2.44	BIRC2
A_33_P3281572	-2.44	CMAHP
A_24_P12539	-2.44	KBTBD2
A_24_P23245	-2.44	NDUFA6
A_24_P63522	-2.44	HMGCS1
A_23_P253412	-2.43	MRPL50
A_23_P204304	-2.43	PTPRO
A_23_P111701	-2.43	GNG11
A_33_P3433873	-2.42	ZWILCH
A_23_P63371	-2.42	TAL1
A_24_P337397	-2.42	ANKRA2
A_23_P66664	-2.42	TADA2A
A_23_P324718	-2.41	SYNJ1
A_23_P25994	-2.41	LGMN
A_23_P131825	-2.41	TNNC2
A_33_P3862375	-2.40	USP45
A_33_P3332112	-2.40	FAS
A_23_P157795	-2.39	CTNNAL1
A_23_P55880	-2.39	ZNF564
A_33_P3290328	-2.39	DCUN1D1
A_24_P173823	-2.39	PBX1
A_23_P72668	-2.39	SDPR
A_24_P99639	-2.39	NEK8
A_24_P360078	-2.39	LRBA
A_33_P3385062	-2.39	HAUS3
A_24_P216654	-2.38	SOAT1
A_23_P218608	-2.38	EIF5B
A_23_P118150	-2.38	ARL6IP1
A_23_P7056	-2.38	SDAD1
A_23_P68472	-2.38	DPM1
A_23_P5742	-2.37	CCDC121
A_24_P14367	-2.37	PTBP1
A_24_P472455	-2.37	ARF6
A_19_P00318418	-2.37	TBXAS1
A_23_P16242	-2.37	ZNF20
A_33_P3323298	-2.36	JUN
A_23_P110569	-2.36	TRIM36
A_23_P251647	-2.36	RFESD
A_23_P56567	-2.36	GEMIN6
A_23_P389102	-2.35	MYO1D
A_23_P75516	-2.35	PPFIA1
A_33_P3371727	-2.35	SAT1
A_23_P124427	-2.34	NEK1
A_23_P26021	-2.34	COPS2
A_23_P69826	-2.34	DHX15
A_33_P3316983	-2.34	BBIP1
A_23_P47904	-2.33	CCDC65
A_32_P194821	-2.33	RPL21
A_32_P96134	-2.33	DPY19L1
A_23_P101351	-2.33	ZNF426
A_32_P205553	-2.33	RPL26L1
A_33_P3295066	-2.33	RPL23P8
A_24_P257579	-2.33	EPB41L4A
A_33_P3381127	-2.33	FAS
A_33_P3378514	-2.32	PDE5A
A_32_P465742	-2.32	PIP5K1B
A_23_P99930	-2.32	TIPIN
A_32_P305888	-2.32	SH3TC2
A_23_P165402	-2.32	SF3B6
A_23_P12526	-2.31	TP53BP2
A_24_P124992	-2.31	PSMA4
A_23_P135857	-2.31	EIF2AK3
A_23_P159839	-2.31	C1GALT1C1
A_24_P44462	-2.31	TPM1
A_33_P3346348	-2.30	TBC1D31
A_23_P16032	-2.30	TRAPPC2B
A_33_P3338928	-2.30	DAB2
A_23_P434809	-2.29	S100A8
A_23_P71537	-2.29	CSPP1
A_33_P3416420	-2.29	ZUFSP

Probe ID	FC (PAX vs Tempus)	Gene
A_33_P3247644	-2.29	MTURN
A_23_P94216	-2.29	LONRF1
A_23_P8763	-2.29	PTPN12
A_24_P652700	-2.28	CEP152
A_23_P169050	-2.28	MRPS28
A_32_P30710	-2.28	RPL23
A_23_P99747	-2.28	CDKL1
A_23_P25974	-2.28	TTC7B
A_23_P319133	-2.28	DNAJC10
A_33_P3384452	-2.28	TFDP1
A_33_P3383029	-2.28	MXI1
A_24_P53282	-2.28	CPD
A_23_P99741	-2.27	CDKL1
A_33_P3322353	-2.27	CAPZA2
A_23_P160567	-2.27	ZMYND12
A_23_P114947	-2.26	RGS2
A_33_P3716128	-2.26	SMC4
A_23_P131240	-2.26	UBXN2A
A_24_P830667	-2.26	RPL21
A_24_P370096	-2.26	ZNF230
A_23_P140434	-2.25	MYO5C
A_23_P146512	-2.25	GOLM1
A_24_P126557	-2.25	RAVER1
A_23_P39799	-2.25	LOXL3
A_24_P47182	-2.25	VCL
A_23_P200001	-2.25	NEXN
A_19_P00318409	-2.25	NEAT1
A_23_P204782	-2.25	MDM1
A_33_P3385983	-2.25	ZNF20
A_32_P234738	-2.25	RPL21
A_23_P134814	-2.25	THAP1
A_33_P3362353	-2.24	ARAF
A_23_P31315	-2.24	CBX3
A_24_P538403	-2.24	ROCK1
A_32_P154342	-2.24	SLCO4C1
A_32_P178945	-2.23	YOD1
A_23_P6836	-2.23	IP6K2
A_33_P3407065	-2.23	KIF2A
A_23_P2683	-2.23	RPAP3
A_24_P926507	-2.23	SLC14A1
A_23_P146050	-2.23	ZFAND1
A_19_P00321073	-2.23	FLJ45950
A_24_P389608	-2.22	PROSER2
A_33_P3386150	-2.22	NRADDP
A_23_P155939	-2.22	ZNF595
A_23_P115885	-2.22	MINPP1
A_24_P346431	-2.21	TNS3
A_32_P182662	-2.21	AIDA
A_23_P98218	-2.20	CWC15
A_33_P3382959	-2.20	CASP8AP2
A_19_P00322409	-2.20	STXBP5-AS1
A_24_P942030	-2.20	VAMP4
A_24_P202139	-2.20	METTL9
A_24_P284584	-2.19	ZNF559
A_23_P391689	-2.19	PET100
A_24_P357518	-2.19	RPL21
A_23_P130089	-2.19	IFT20
A_23_P55256	-2.19	ZNF652
A_23_P82523	-2.19	ABCB1
A_23_P213754	-2.19	PAIP2
A_23_P134925	-2.19	BNIP3L
A_23_P22096	-2.19	PTK2
A_23_P128940	-2.18	VCPKMT
A_23_P200216	-2.18	MAGOH
A_33_P3249534	-2.18	NEFM
A_19_P00813085	-2.18	lnc-SNURF-1
A_23_P122615	-2.17	PNISR
A_32_P166693	-2.17	HEG1
A_24_P641130	-2.17	PRRC2A
A_23_P338519	-2.17	NKIRAS1
A_23_P214360	-2.17	IRF4
A_33_P3256773	-2.16	INIP
A_33_P3636080	-2.16	MLH3
A_33_P3337019	-2.16	LOC728975
A_23_P82748	-2.16	ENY2
A_23_P81934	-2.15	C6orf25
A_23_P259135	-2.15	NCOA6
A_23_P389118	-2.15	ANO6
A_33_P3282898	-2.15	TLN1
A_24_P418418	-2.15	RPS17
A_24_P12065	-2.15	CCNG2
A_24_P341187	-2.14	GBA2
A_24_P119545	-2.14	ITPKB
A_23_P113701	-2.14	PDGFA

Probe ID	FC (PAX vs Tempus)	Gene
A_23_P43425	-2.14	C9orf40
A_23_P250800	-2.14	ST3GAL6
A_24_P287941	-2.14	PSMC3IP
A_33_P3318581	-2.13	PLOD2
A_23_P64913	-2.13	PDE6H
A_33_P3279515	-2.13	LOC729451
A_23_P121806	-2.13	ENOPH1
A_23_P204324	-2.12	DNM1L
A_23_P20832	-2.12	SPTAN1
A_19_P00322407	-2.12	STXBP5-AS1
A_24_P200162	-2.12	HIGD1A
A_24_P147252	-2.12	ZNF23
A_23_P62605	-2.12	RPL11
A_19_P00809873	-2.12	ND1
A_23_P209619	-2.11	ATL2
A_33_P3370404	-2.11	PANX1
A_23_P98995	-2.11	CALCOCO1
A_24_P126417	-2.11	USP34
A_33_P3381777	-2.11	TREML1
A_23_P4161	-2.11	ARSG
A_24_P71373	-2.11	SLC9A1
A_24_P940166	-2.10	PAPSS2
A_23_P358221	-2.10	UBXN7
A_23_P311885	-2.10	L3MBTL3
A_23_P426472	-2.10	ZNF45
A_24_P280378	-2.10	AQR
A_23_P163099	-2.10	POLE2
A_33_P3417150	-2.10	P2RY1
A_32_P140139	-2.10	F13A1
A_33_P3333317	-2.09	OPTN
A_23_P35916	-2.09	ATM
A_23_P152906	-2.09	ALOX12
A_23_P53467	-2.08	IKBIP
A_23_P54116	-2.08	DAAM1
A_23_P80062	-2.08	TAF4
A_24_P935009	-2.08	CRKL
A_23_P253586	-2.08	DOPEY2
A_24_P310630	-2.08	UPF3B
A_23_P81463	-2.08	DHX29
A_23_P25873	-2.08	WDHD1
A_23_P151321	-2.07	RPL6
A_32_P162150	-2.07	TAB3
A_23_P427217	-2.07	JMJD1C
A_23_P140328	-2.07	NEMF
A_19_P00808408	-2.07	MBD5
A_24_P134816	-2.06	BCL9L
A_33_P3344169	-2.06	DYX1C1
A_23_P328237	-2.06	SPTY2D1
A_33_P3273136	-2.06	MCUR1
A_23_P42322	-2.06	COL11A2
A_23_P117558	-2.05	FKBP3
A_19_P00317108	-2.05	LINC00657
A_23_P2725	-2.05	RPL21
A_23_P163143	-2.05	ACYP1
A_23_P254031	-2.05	TTF1
A_23_P55948	-2.05	PRR12
A_24_P313186	-2.04	CALM1
A_23_P155147	-2.04	ZBED4
A_23_P140290	-2.04	RTN1
A_23_P137073	-2.04	ZMYM3
A_33_P3380682	-2.04	TBPL1
A_23_P67339	-2.04	RCN3
A_19_P00811196	-2.03	APTR
A_33_P3416037	-2.03	FAM96A
A_24_P38815	-2.03	TPP1
A_23_P143902	-2.03	P2RY12
A_33_P3284858	-2.03	LOC729867
A_33_P3310780	-2.03	CTTN
A_23_P44768	-2.03	TBK1
A_24_P374382	-2.03	TOP1P2
A_33_P3316639	-2.03	CHMP3
A_19_P00804215	-2.03	FAM228B
A_24_P227091	-2.03	KIF11
A_24_P416289	-2.02	KIAA0195
A_23_P207666	-2.02	USP32
A_23_P46315	-2.02	DENND2C
A_23_P210538	-2.01	ELMO2
A_24_P226962	-2.01	KIAA0368
A_23_P110643	-2.01	CDKL3
A_24_P321634	-2.01	ZMYM5
A_24_P55465	-2.00	MTPN
A_33_P3304983	-2.00	PRKAR2B
A_33_P3407606	-2.00	MSN
A_19_P00321333	-2.00	NEAT1
